# Towards the Physics of Calcium Signalling in Plants

**DOI:** 10.3390/plants2040541

**Published:** 2013-09-27

**Authors:** Teresa Vaz Martins, Matthew J. Evans, Hugh C. Woolfenden, Richard J. Morris

**Affiliations:** Computational and Systems Biology, John Innes Centre, Norwich Research Park, Norwich NR4 7UH, UK

**Keywords:** mathematical modelling, calcium, Ca^2+^ ion channels, systems biology, symbiosis, tip growth, stomata, circadian rhythms, oscillations, signal transduction

## Abstract

Calcium is an abundant element with a wide variety of important roles within cells. Calcium ions are inter- and intra-cellular messengers that are involved in numerous signalling pathways. Fluctuating compartment-specific calcium ion concentrations can lead to localised and even plant-wide oscillations that can regulate downstream events. Understanding the mechanisms that give rise to these complex patterns that vary both in space and time can be challenging, even in cases for which individual components have been identified. Taking a systems biology approach, mathematical and computational techniques can be employed to produce models that recapitulate experimental observations and capture our current understanding of the system. Useful models make novel predictions that can be investigated and falsified experimentally. This review brings together recent work on the modelling of calcium signalling in plants, from the scale of ion channels through to plant-wide responses to external stimuli. Some *in silico* results that have informed later experiments are highlighted.

## 1. Introduction

Numerous stimuli lead to changes in calcium concentrations that regulate plant responses, so obtaining insights into how plants adapt to their environment often requires understanding the processes that govern calcium levels within and between compartments. Many processes are, however, so complex that our understanding is hindered by a breakdown of intuition. Such complexity can arise already with three or more variables for non-linear interactions, as are typical for biological systems. Building simplified mathematical models that capture the key characteristics of the system under study is in such cases a fruitful approach for explaining and understanding the observed behavior, as well as providing hypotheses for the underlying mechanisms.

In this contribution we review mathematical and computational approaches to calcium signalling in plants. We present a systems biology dissection of calcium signalling processes with some selected examples that demonstrate how this approach has helped to unravel complex phenomena and guide further experiments. Whilst the review aims at being comprehensive it is not exhaustive and we apologise to those authors whose work is not adequately represented. A number of common methodological techniques are summarised and we provide key equations and list some popular software packages. The review concludes with an outlook of future challenges and application areas.

## 2. Calcium by Numbers

### 2.1. Calcium Biochemistry

Calcium is the fifth most abundant element by mass in the Earth’s crust and in the human body. It is an alkaline earth metal with an atomic number of 20 and a standard atomic mass of 40.078 u. Alkaline earth metals all have 2s electrons in their outer shell and their relatively low ionisation energies lead to the formation of doubly charged, filled-shell cations. The first two ionization energies of calcium are 590 kJ/mol and 1,145 kJ/mol whereas the third ionization energy is much higher at 4,912 kJ/mol. The common form of calcium in solution is therefore Ca^2+^ which has an effective ionic diameter of 2 Å. In seawater Ca^2+^ is the fifth most abundant dissolved ion by mass at around 400 mg/L or 10 mM. The Ca^2+^ concentration of drinking water varies from about 1 to 135 mg/L [1], corresponding to a range of 0.03 to 3.38 mM.

The flexibility of Ca^2+^ to form chemical bonds with different coordination numbers and geometries gives it the ability to form interactions with membranes, small molecules and proteins. An analysis of small molecule and protein crystal structures shows that Ca^2+^ generally binds to oxygen and that the preferred coordination numbers range from 6 to 8 [2]. In particular, Ca^2+^ binds to phosphate groups to form insoluble compounds thus rendering high cytosolic Ca^2+^ concentrations toxic to the cell. This impact of Ca^2+^ on phosphate groups is consistent with the observation of Williamson [3] and Tazawa *et al*. [4] who established that cytoplasmic streaming, which requires ATP, was dependent on a very low Ca^2+^ in the order of 0.1 µM. If the concentration was elevated to 1.0 µM, cytoplasmic streaming was decreased by 20%, and when Ca^2+^ was increased to 10 µM, the streaming was inhibited by more than 80%. Similar observations can be expected for other ATP-dependent processes. The presence of Ca^2+^ binding molecules as a means of reducing the concentration of free Ca^2+^ would therefore be of advantage to the cell.

**Figure 1:**
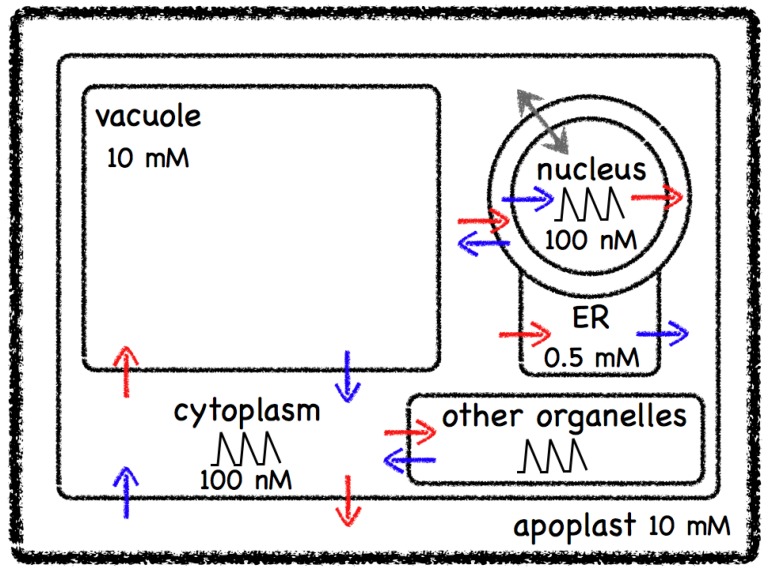
A schematic view of a plant cell showing some of the main calcium stores and cellular compartments with their approximate free calcium ion concentrations. Calcium channels are shown as blue arrows, pumps are shown as red arrows, whilst the grey double arrow depicts diffusion through the nuclear pores.

Uniprot [5] lists over 400 proteins with a gene ontology molecular function of calcium ion binding (GO:0005509) in *Arabidopsis thaliana* and TAIR [6] lists 520 loci with 622 distinct gene models out of a total of 31,845 genes, so in the order of 2% of the genes are involved in Ca^2+^ binding. Lowering of the free cytosolic Ca^2+^ through buffering/binding is assisted by specialized transporters, which can move Ca^2+^ against a concentration gradient between compartments. As illustrated in Figure 1, cells maintain a steady state Ca^2+^ concentration of about 100–200 nM in contrast to the Ca^2+^ concentration in the extracellular space at about 1–10 mM, approximately the value seen in water. Such a large concentration difference allows for rapid signalling responses through the opening of specialised calcium channels in the membranes separating compartments.

The release of Ca^2+^ from internal stores, such as the endoplasmic reticulum or the vacuole, gives rise to transient elevations in Ca^2+^ from about 0.1 µM to 1 µM. The characteristics of such transients vary and can become oscillatory with periods from seconds to hours. The localised free cytosolic Ca^2+^ concentration varies in duration, amplitude, frequency and spatial distribution, and these observations led to the “Ca^2+^ signature” hypothesis [7], which states that signal information is encoded by a spatio-temporal pattern of cytosolic Ca^2+^ concentrations. Changes in the concentration of free cytosolic Ca^2+^ can be perceived by calcium binding proteins that can lead to the activation of different cellular programmes. It has been suggested that the need to reduce the toxic effects of high free Ca^2+^ concentrations drove the evolution of components that could then be taken advantage of as calcium signalling machinery [8].

These factors have led to Ca^2+^ becoming one of the most important signalling ions in higher eukaryotes and a ubiquitous second messenger in plants [9] and animals [10,11]. Ca^2+^ can transduce both intercellular and intracellular signals and is involved in nearly all aspects of plant development as well as participating in many regulatory processes.

### 2.2. Calcium Maths

A frequent goal of developing mathematical models of processes involving calcium is to aid our understanding of how calcium ion concentration changes are generated as a function of the individual components. A large class of calcium signalling models consists of systems of ordinary differential equations (ODEs) describing fluxes between different compartments. Ignoring spatial effects, which is equivalent to assuming that the calcium concentration, *c*, is homogeneous inside each compartment, results in the ODE:

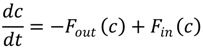
(1)
which describes the change of the concentration with time, where 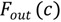 and 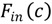 represent the sum of all fluxes out of and into the compartment respectively. Describing the changes in different compartments, results in a system of such equations. In some cases it is useful to model explicitly the time lapse before changes occur, such as in gene translation, and this can be accounted for using a delay parameter, leading to a variant of the above approach known as delay differential equations (DDEs). Spatial models are based on partial differential equations (PDEs) and include the additional term, 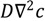,

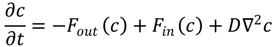
(2)
which accounts for the diffusion of calcium in the (now partial rather than total) derivative of the calcium concentration. Within this framework, relevant compartments need to be identified for the process under study and the various fluxes between them need to be understood. Selected examples will be considered in the following sections. Once the compartments, the machinery and the resulting fluxes have been identified, established numerical procedures can be employed to integrate the differential equations and to deliver the evolution of the calcium concentration and fluxes.

This concentration-based approach is popular due to the available numerical techniques for integrating ODEs but it is also plausible based on the number of ions and their diffusibility in large compartments. For a [Ca^2+^] of 100 nM a cell with a cytoplasmic volume of 200 μm^3^ would contain about 12,000 ions, a nuclear volume of 100 μm^3^ would have around 6,000 ions but a small organelle of 1 μm^3^ would only hold around 60 calcium ions. Given these numbers and the locality of calcium signatures, low copy number effects are likely to be relevant at physiological Ca^2+^ concentrations in smaller compartments [12] as the number of calcium ions involved decreases below the continuum limit of ordinary differential equations [13]. Monte Carlo techniques such as Gillespie’s algorithm [14] or variants thereof provide easy to use solution strategies for such descriptions that track particle numbers based on discrete events.

It is thus fairly straightforward to write down a model of a system and to integrate the equations. Using feedback mechanisms and/or time delays, oscillations are relatively easy to establish and with the power of optimisation techniques these can often readily be made to reproduce experimental data. The challenge is therefore to suggest models that say something interesting about the biology and to make testable predictions.

## 3. Models for the Calcium Machinery

**Figure 2:**
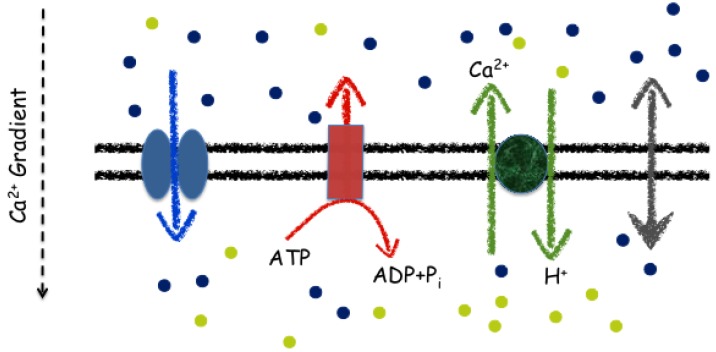
Schematic diagram of different mechanisms of passive and active transport. Ca^2+^ (blue dots) is transported across the membrane down its electrochemical gradient by the gated ion channel (blue arrow, left) and by the leak channel (grey arrow, right, with fuller arrowhead pointing to the side with a lower Ca^2+^ concentration ) Both the ATP-dependent pump (red arrow) and the Ca^2+^-H^+^ antiporter (green arrows) transport Ca^2+^ against its electrochemical gradient, which requires energy. The ATP-dependent pump uses the energy derived from ATP hydrolysis to ADP and inorganic phosphate (P_i_), while the calcium exchanger uses the free energy released from the movement of H^+^ (green dots).

The dynamical properties of Ca^2+^ signals depend in the first instance on the machinery that regulates Ca^2+^ fluxes between cellular compartments (see Figure 2). The activation of calcium-release channels leads to an influx of Ca^2+^ while the subsequent restoration of basal non-toxic calcium levels and the replenishment of Ca^2+^ stores requires pumps to transport calcium against the concentration gradient. The Nernst equation,

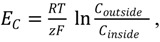
(3)
can be used to determine the voltage across the membrane, *E_C_*, termed equilibrium or reverse potential, that would maintain the ion concentrations, *c_outside_* and *c_inside_*, in steady state. Here *R* = 8.31 J·K^−1^ mol^−1^ is the universal gas constant, *T* is the absolute temperature in Kelvin, *z* is the valence of the ion (*z* = 2 for Ca^2+^) and *F* = 9.65 × 10^4^ C mol^−1^ is the Faraday constant. At T = 290 K, a calcium concentration of 10 mM outside the cell and of 100 nM inside the cell, results in an equilibrium potential across the plasma membrane of 144 mV. The electrochemical driving force is given by the difference between *E_C_* and the membrane voltage, *v*, and for a single channel, the ohmic current, *I_C_*, is given by

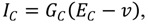
(4)
where *G_C_* is the channel conductance. Proton ATPases generate a voltage of about −150 mV across plant plasma membranes, resulting in an electrochemical driving force for Ca^2+^ of about 300 mV.

Channels and pumps can exist in an open or closed configuration, and plant survival depends on maintaining the correct configurations at the right time. The term “gating” is used to describe the mechanism by which the channel/pump controls the transition between the open and closed states. Gating permits the maintenance of large concentration differences with the exterior of the cell or internal stores during resting periods, and the concerted release of calcium during signalling events. Channels can be classified as voltage-gated, ligand-gated, mechanically sensitive, store-operated, light- or temperature-activated, among others. This is a broad classification, as the activation of channels may require a complex regulation by multiple stimuli.

To obtain the macroscopic current density the individual conductance is multiplied by the fraction of open channels, which is commonly given by a semi-empirical function with a steep non-linear dependence on the activation variable. The Hodgkin-Huxley activation function [15] is typically used for voltage-gated channels. The channel current, *I_C_*, is given by

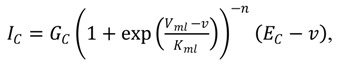
(5)
where *n* is the number of identical and independent activation gates, *V_ml_* is the half-maximal activation of voltage gated channel, and *K_ml_* is a constant in the scaling function. For a ligand-gated channel a Hill function [16] may be used and the channel current is

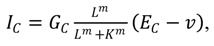
(6)
where *L* is the ligand concentration, *K* is the ligand concentration producing half maximal response and *m* is the Hill coefficient that, in principle, represents the number of binding sites. Positive cooperativity is defined to occur when *m* exceeds one and negative cooperativity when *m* is less than one. Both Equations (5) and (6) contain the steep non-linear dependence mentioned earlier.

Although the mathematical description of channel and pump activation is similar, their relation to the electrochemical force, *E_C_* − *v*, is fundamentally different. Ion transport through channels is driven by the electrochemical force whereas pumps must overcome the potential difference by either harnessing the energy released from ATP hydrolysis (ATPases) or by coupling the transport of one ionic species down its electrochemical potential to drive the thermodynamically uphill transport of another species (exchangers). The free energy released from the conversion of ATP to ADP under standard conditions (1 M ATP, 1 M ADP, 1 M phosphate, 55 M H_2_O, T = 298 K) is about −30 kJ/mol. Cells operate far from these conditions with ATP/ADP ratios often around 5 to 10 and mM phosphate concentrations. The resulting free energy for ATP under these conditions is about −50 to −60 kJ/mol. The free energy required to drive Ca^2+^ against a concentration ratio of 10^5^ is zF (*E_C_* − *v*) = 56.7 kJ/mol.

We begin with simple flux models where the consideration of many channels leads to an all-or-none representation of activation functions, and then focus on kinetic models that probe the activation steps of a single channel.

### 3.1. Fluxes: Ligand- and Voltage-Gated Calcium Release Channels

The capacity for auto-regulation—a distinctive property of calcium amongst ions [17]—underlies several models of Ca^2+^ oscillations. Calcium release regulates further calcium release either directly by the calcium-induced-calcium-release (CICR) process [18,19], or indirectly by activating pumps [20] or other essential cation channels acting in parallel [21,22].

One of the earliest models [18,19] of calcium signalling in plants was motivated by the observation that in the unicellular green alga *Eremosphaera viridis*, the presence of Sr^+^ or caffeine induces repetitive [Ca^2+^] spiking in the cytosol. The model consists of a system of ODEs that describes calcium fluxes between the cytosol, the exterior and an indeterminate internal store. Since the vacuole is the largest calcium store it could be seen as a natural choice, but, perhaps counterintuitively, it is its size that excludes it. The clue to the store identity is given by the fact that at least two variables are required for self-sustained repetitive spiking [13,19,23]. Therefore, considering that the high vacuolar calcium levels are unlikely to fluctuate significantly enough, the authors predict that the relevant internal store is the small endoplasmic reticulum (ER). Experimental evidence suggests the involvement of a ryanodine receptor type of Ca^2+^ channel, which in animals is activated by Ca^2+^, and that Sr^+^ is required for the initiation of calcium oscillations. Therefore, the model assumes that cooperative Sr^+^ and Ca^2+^ binding activates the channel—a Hill function (see Equation (6)) represents this type of cooperative activation in a phenomenological way. Fluxes result from the interplay between calcium release and reuptake by Ca^2+^-ATPase pumps whose activity is proportional to cytosolic calcium, while the frequency and amplitude of calcium oscillations depends on the Sr^+^ dose. To be consistent with observation of a calcium efflux from the ER even when Ca^2+^ and Sr^+^ are very low, Bauer *et al*. [18] also include a leak term, or non-gated channel. The model reproduces the generation of repetitive calcium spiking, while making predictions about the nature of the channels and pumps and the identity of the internal store.

Focussing on a minimal model [23], Bauer *et al*. [18] aimed to extract only the key players needed to reproduce Ca^2+^ transients. For example, experimental evidence shows that the addition of Sr^+^ induces a transient hyperpolarisation of the plasma membrane, which accompanies cytosolic calcium increase. While hyperpolarisation is attributed to the opening of a plasma membrane K^+^ channel activated by calcium, the variation in the membrane potential does not seem to have a significant influence on calcium release. Therefore the model does not include the potassium channel: the perturbation of the calcium electrochemical driving force would tend to change the magnitude of the fluxes, but the interplay between the ligand-gated calcium-release-channel and pump ensures that the final balance of fluxes is not disrupted.

In contrast, a similar potassium channel activated by calcium binding is a key element in a model proposed by Granqvist *et al*. [22] and Charpentier *et al*. [21]. A K^+^ channel (DMI1 [24]), a calcium-release channel and a Ca^2+^-ATPase are all essential transporters present in the nuclear membrane during plant symbiosis in *Medicago truncatula.* A crucial difference with the previous model [18] is that here the calcium-release channel is voltage-gated (see Equation (5)), activated by the depolarisation of the nuclear membrane. The proposed scenario contains all the minimal elements for sustained membrane voltage oscillation [25,26]. In particular, the different Ca^2+^ and K^+^ reverse potentials guarantee the distance from equilibrium, and the calcium channel provides positive self-coupling. Calcium influx into the nucleus increases the membrane voltage, which increasingly activates the channel—a related proposal that included a hyperpolarisation activated calcium channel could only result in a single spike [27]. Oscillations of calcium concentration open and close DMI1 leading to a periodic K^+^ efflux, and the resulting changes in the membrane potential are crucial to guarantee sustained nuclear Ca^2+^ oscillations.

### 3.2. Gating in Steps

The models described above [18,21,22] all accurately reproduce the shape of the calcium transients, with the use of phenomenological activation functions. Kinetic mechanistic models of gating or permeation [28,29,30,31] can be used to bypass this long acknowledged limitation [32]. With an explicit representation of single ion-channel states, such models propose a direct link with the channels’ structural identity, by relating gating steps or binding sites with conformational states with a clear biophysical meaning.

Tidow *el al*. [20] studied a plasma membrane ATPase in *Arabidopsis thaliana*. Such Ca^2+^ pumps are activated by calcium-loaded calmodulin (Ca^2+^-CaM) and crystallographic studies uncovered two autoinhibitory binding sites, instead of one as was expected. It is a mathematical model of kinetic rate equations that elucidates the physiological importance of this discovery, by relating the successive relief of the three autoinhibitory sites to the calcium concentration. It shows that the pump is inactive below a basal Ca^2+^ concentration; it gets ready for activation at that level, and rapidly increases its activity above the basal level when the two binding sites are occupied. This three-step process leads to an abrupt increase of the pumping activity for high calcium concentrations, and assures the stability of non-toxic basal levels. Therefore, the model demonstrates that the different, experimentally found, conformational states, are essential to regulate calcium currents.

In another system, the model of Pottosin *et al*. [33] starts from the observation of regulated cation currents from a slow vacuolar channel to predict the existence of distinct gating steps. The vacuole is a crucial organelle for plant homeostasis, which regulates cytoplasmic ion concentration, although the significance for Ca^2+^ release is still uncertain [34,35]. Among the vacuolar channels [36], the large population of the non-selective and highly-conductive slow vacuolar [37] (SV) channels would rapidly lead to unsustainable currents if its activity was not restricted. While cytosolic Ca^2+^ promotes the opening of SV channels, vacuolar calcium is essential to down-regulate the activity of SV channels to physiological levels.

Pottosin *et al*. [33] observe that the effect of Ca^2+^ on the current of a single open channel is comparable to that of another divalent cation, Mg^2+^. However, Ca^2+^ has a much more pronounced effect on the macroscopic current, suggesting the existence of two independent binding sites: permeation [38] is reduced upon either Mg^2+^ or Ca^2+^ binding, while channel gating involves another binding site that is most selective for Ca^2+^. The authors seek to clarify how vacuolar Ca^2+^ inhibits the macroscopic SV current. They apply the patch-clamp technique to measure the currents from the vacuoles of sugar beet (*Beta vulgaris* L.) taproots, and observe a biphasic dependence of the SV current on the membrane potential. In line with the flux models of the previous subsection, this could possibly be explained by various voltage dependencies of possible pumps and cation channels. Following a different approach, the authors concentrate instead on the channels gating mechanisms. They propose that SV channels pass through two different closed conformations before opening, and use Boltzmann statistics to assign the probability of each state and their transitions. Charge parameters indicate the steepness of the voltage-dependence for each transition. Binding of different numbers of Ca^2+^ ions stabilizes the closed conformations and shifts the activation towards more positive voltages. An important result is that multiple ions bind to the first closed state, because multiple binding can explain how SV channels can be strongly down-regulated within the range of physiological calcium levels. Additionally, the authors identify the transition to the open state as the rate-limiting step and observe that vacuolar Ca^2+^ decelerates the channel activation. Since deceleration means an increase in the energy-barrier for that transition, this implies that binding can significantly alter the structure of the channels, which is an important dynamical dimension of gating often disregarded by the simplest kinetic models.

The recent characterisation of complex channels in plants, such as cyclic-nucleotide gated channels (CNG) [39,40] will probably lead to the expansion of the current gating framework. CNG gated channels are mainly activated by cyclic nucleotide binding to four units, but they are also weakly dependent on the voltage, and the likely complex interaction between these multiple sources of activation has yet to be modelled. Modelling in animal systems is already exploring how the binding of one unit may induce a conformational change which alters the affinity of subsequent bindings, proposing schemes such as induced fit or allosteric control [41], which further introduces a dynamical character in the gating processes.

### 3.3. The Basic Machinery: Autonomous Regulation of Calcium Levels in Organelles

The study of compartmentalized signals provides the ideal setting to identify a minimal signalling toolkit in a closed system and also to show how the effects of an individual signalling component must be framed in the context of the entire system. It is also an area of basic importance in the field of calcium signalling in plants: compartmentalisation of calcium signalling in different cellular organelles can permit the independent control of specific functions, such as gene transcription inside the nucleus [42,43]. Autonomous nuclear calcium signalling is still controversial due to the limitations of experimental techniques [44,45,46,47].

Following the discovery that an impermeable nucleus of BY-2 tobacco cells responds to various abiotic stresses by an increase in calcium levels [48], Brière *et al*. [49] sought to identify the minimal components required for the generation of autonomous nuclear calcium transients. They present an ODE model describing fluxes between the nucleus and the nuclear envelope (NE) considered as a calcium store [50]. The model is biologically based on evidence [48] that shows that various stimuli—temperature, pH variation or mechanical stress—evoke a single calcium transient rise followed by a slow restoration of basal levels, and that successive stimulations lead to a train of sustained Ca^2+^ peaks. Furthermore, experiments conducted in parallel with the development of the model show that the nucleus is impermeable to a calcium bath.

Whereas the increase in nuclear calcium concentration is assumed to result from the influx by non-specific channels localized in the inner nuclear membrane, the restoration of basal calcium levels is unexplained at the start. Buffers reduce the amount of free Ca^2+^, but their typically fast association and dissociation rates are incompatible with the slow kinetics of calcium decrease. Therefore, the authors predict the existence of yet unidentified pumps that would drive Ca^2+^ back into the NE. Furthermore, pumps enable a response to successive stimuli by refilling the calcium stores—something that buffers would not do. In fact, a later work [51] identified pumps in the inner nuclear membrane of *Medicago truncatula*, providing a confirmation—albeit in a different model plant—that the nucleus has autonomous minimal signalling machinery composed of pumps and channels*.*

While the model of Brière *et al*. [49] considered an impermeable nucleus, in other systems observed relations between cytosolic and nuclear oscillations are difficult to explain with an isolated nucleus [51]. During legume symbiosis, apparently synchronised perinuclear oscillations are observed on both sides of the nuclear envelope. However, the resolution of confocal imaging is insufficient to exclude a slight delay, and thus a possible cytosolic or nuclear origin of the perinuclear oscillations. Although the extent to which the nuclear envelope is permeable to calcium is unclear [49,52,53,54,55,56], if the pores were permeable, it might be assumed that passive diffusion across the NE would eventually synchronize calcium oscillations. However the model developed by Capoen *et al*. [51] showed that this need not be the case; the large permeability of the individual pores does not imply a large permeability of the entire nuclear envelope.

Ca^2+^ signalling is a result of complex, nonlinear interactions that cannot be properly understood by the analysis of the separated components alone. The authors start from the assumption that calcium signalling originates on one side of the NE and propose a spatial model where inner and outer nuclear membranes are connected by pores that allow free passage of Ca^2+^. They place calcium-permeable channel clusters only at the cytosolic side of the nuclear membrane, to investigate a possible cytosolic origin of nuclear calcium oscillations. An adaptation of the fire-diffuse-fire model [57,58] with linear uptake [59] is chosen to study calcium diffusion over the surface of the nuclear membranes and across the nuclear envelope. As their dimensions are very small compared to the area of a nuclear membrane, both channel clusters and pores are considered as discrete sources of calcium and placed at uniformly random locations in the inner and outer nuclear membranes. This is an appropriate framework to capture the calcium spatial microdomains that are observed during plant symbiosis [60]; by the CICR mechanism, Ca^2+^ released by a channel diffuses to activate other channels in a sequence of release events. High Ca^2+^ levels inhibit further release, resulting in a refractory period that affects the period of oscillations.

Simulations [51] show that even if calcium can freely diffuse across the nuclear membranes, oscillations cannot be transmitted across the NE. Even choosing an unusually high pore density, the fraction of the nuclear membrane surface occupied by pores is very small, and besides, an important part of the calcium released by a channel is pumped back into the NE before it reaches the nearest pores. To conclude, if calcium release from the inner nuclear membrane is observed and it cannot originate in the cytosol, the nucleus must have its own signalling machinery. Therefore, this work provides confirmation of the basic assumption in Brière *et al*. [49] but suggests the need for an as yet unidentified source of coordination between cytosolic and nuclear calcium signalling. The authors propose that the NE is the locus of that source.

Oscillations generally result from a balance of positive and negative feedback. Due to the lack of positive feedback in the model of Brière *et al*. [49] repetitive spiking can only result from repetitive stimuli. Whereas in Capoen *et al*. [51] self-sustained oscillations can be achieved via CICR feedback. Despite this and other differences, both models [49,51] assumed that the NE is the source of calcium and nuclear calcium is released near the interface with the outer nuclear membrane. In the tobacco nuclei system studied by Brière *et al*. [49], this scenario made autonomous nuclear oscillations look improbable—and required the explanation that pores are impermeable in the system. On the contrary, in *Medicago* symbiosis, permeable nuclear pores provided a ready explanation for synchronised nuclear and cytosolic oscillations of similar amplitude—that was nevertheless questioned by modelling [51].

In relation to the independence of nuclear calcium signalling it should be mentioned that possible nuclear invaginations could target calcium to specific locations within the nucleus thereby enhancing its autonomy. Modelling such complex geometries requires suitable computational approaches that are still pending in plants. Models in the field of animal studies offer interesting perspectives that combine experimental confocal data, *in silico* 3D reconstruction of the detailed morphology, numerical multi-grid solvers and mathematical approaches, e.g., finite element method or others [61,62,63,64]. The transfer and adaptation of these techniques to plants will shed light into domains inside compartments that remain obscure by models that can only deal with simple shapes.

## 4. Calcium and Temperature

As the models in the previous section demonstrated, calcium signalling requires, at the very least, channels and pumps. Since the behaviour of pumps differs from channels in that pumps transport ions thermodynamically uphill instead of downhill [19], temperature seems to be the basic variable so far ignored in our presentation.

Plants are threatened by [65] but also contribute to [66] changes in climate. Since they cannot move as the weather changes, the ability to respond to variations of temperature is critical for plant survival as the seasons pass or the day turns into night [67]. A change in calcium levels is one of the earliest responses to cold [68,69,70]. However, as most biological processes are affected by temperature, its overall effect is neither intuitive nor specific and it is a challenge to model the mechanisms behind plant acclimation. Ultimately, the key question is not how temperature affects calcium levels but what, if any, is the role of calcium in the plant adaptation to temperature. It is important to ascertain whether the calcium response is upstream of the temperature signalling pathways [71] and therefore, as it has been considered [72], the primary temperature-sensing event [73]. So far definitive answers have proved elusive, however modelling has uncovered various effects of temperature that directly impact on the calcium response.

### 4.1. The Search for Primary Temperature-Sensing Events

Plants respond to a cold shock by a transient rise in cytosolic calcium levels [74], and to study the mechanisms behind this reaction, Plieth proposes [75] a one-compartment model of the fluxes across the plasma membrane. The main hypothesis is that the temperature sensor in plants is a calcium release channel whose activity increases sharply with the cooling rate, while the activation of a Ca^2+^ATPase explains two experimental observations: sensitisation and desensitisation. Thus, sensitisation, or an increase of the calcium response to cooling at lower temperatures, is attributed to a known [76] exponential increase of the pump enzymatic activity with the absolute temperature. To counteract the inhibition of pump activity with cold and explain the attenuation of the calcium response with time of exposure to cold, desensitisation is modelled by an increase of the number of active pumps with calcium levels. This simplified model reproduces qualitatively well the experimental data, supporting (without confirming) the suggestion that the calcium channel is a primary thermal sensor.

Plieth [75] uses a phenomenological temperature-dependent function to model how the calcium channel is activated by cooling. White *et al*. [77] aim to explain the same experimental results [74] without hypothesizing temperature gating. The authors [77] implicate voltage-gated calcium channels in the response to cooling, with a focus on the “maxi” cation pore in the plasma membrane of rye (*Secale cereale* L.) roots. This is a depolarisation-activated channel permeable to various cations, and in particular to Ca^2+^. The Eyring rate theory [78], [79] represents the movements of ions inside the channels as a sequence of stochastic jumps across temperature-dependent high energy barriers separating energetically favourable binding sites. White *et al*. [77] propose a permeation model with three barriers and two binding sites (known as 3B2S) [77,80,81,82], and combine it with a gating-kinetics model with empirically determined voltage-dependent transition rates. This allows them to determine the calcium released by the channel and observe that it is similar to the cytosolic Ca^2+^ influx observed after a cold shock. These voltage-gated channels contribute to a perturbation of the membrane potential elicited by rapid cooling, called a slow action potential (SAP). Since in turn the SAP opens voltage-gated channels, cooling indirectly activates calcium-release-channels, thus amplifying the initial thermodynamic effect of temperature.

While in Plieth [75] temperature is an explicit variable, other authors propose an indirect way to access its influence by matching the simulated calcium profiles with the experimentally observed Ca^2+^ response to temperature. The parameter combination that corresponds to the best fit, implicitly identifies the key process regulated by temperature. This is the approach adopted by Bose *et al*. [83]. The authors show that different proportions of ATPases to CAX (Ca^2+^ exchangers) result in different calcium signatures that match experimentally observed responses to various types of stress, including cold. Therefore, without establishing an explicit dependence between different efflux systems and temperature, this suggests to look into the different efflux systems when trying to understand the response of plants to cold.

Brière *et al*. [49] follow a similar philosophy, focusing on the nucleus of BY-2 tobacco cells. Evidence shows that under alkaline or neutral pH conditions, the nuclear calcium concentration increases with the temperature of the medium, while a cold shock results in a drop of nuclear calcium levels. By varying parameters, they show that the rising calcium concentration with increasing temperature is best reproduced by an increased influx: the authors suggest that the involved channels may be analogous to the TRP channels that in mammalian cells are the primary sensors of temperature [84,85], although interestingly, recent evidence in *Arabidopsis* [86,87,88] has implicated plasma membrane cyclic-nucleotide gated channels in the perception of heat shocks. On the other hand, a decrease of calcium concentration following a cold shock in alkaline conditions appears to result from an increase in the buffering capacity of the nucleoplasm caused by a rapid reduction of the buffers dissociation constant. It seems natural to wonder if this drop of nuclear calcium levels could lead to an increase of cytosolic calcium, as in the system considered by Plieth [75]. However, this tobacco cell nucleus appears to be impermeable to calcium. Nevertheless, it would be interesting to extend the model of Plieth [75] to incorporate buffers and try to replicate a similar effect as in Brière *et al*. [49]. In fact, Plieth [75] chose to neglect the presence of buffers, arguing that they could only smooth transitions, but not affect the influx-efflux balance. In contrast, the following model by Liu *et al*. [89] finds that changes in the buffering capacity do not influence the response to temperature. However, the origin of this discrepancy with Brière *et al*. [49] is unclear, since the authors [89] use a different mathematical model and refer to a different system.

### 4.2. The Many Elusive Effects of Temperature

Liu *et al*. [89] combines experimental analysis and mathematical modelling to investigate the role of the vacuolar and cytosolic calcium pools in the generation of calcium signatures elicited by temperature changes. They set up a very comprehensive system of ODEs to study fluxes across the membranes, incorporating a variety of transporters: calcium channels, ATPases, symporters and antiporters, a Cl^−^ channel, and K^+^ outward and inward rectifying channels. The influence of temperature is felt at many levels, from the kinetics of all transporters to the ADP/ATP ratio and therefore the ATPase pump activity [90]. At steady state with constant temperature the pools are isolated, but when the temperature changes, the ions equilibrium potential varies, and the cytosol exchanges ions with the vacuole. The ion flow affects the voltage of the membranes and thereby the opening of the channels. The shape of the calcium signature is determined by CICR at the vacuolar membrane, and by the transport of four ions from the cytosol to the vacuolar pool. The model correctly predicts a rise in calcium concentration in response to temperature reduction, especially when the reduction is sharp.

Results are mostly in agreement with Plieth [75], but the model by Liu *et al*. [89] is an example of an integrative approach that tries to capture most of the known important transporters and effects, being considerably more detailed and in that sense more realistic. The downside of this approach is the vast number of parameters it demands; different combinations of parameters can produce similar results, and moreover, in plant systems many values are unknown and taken from the more mature field of animal studies.

It is somewhat disappointing to begin this section with a model [75] that suggests that the calcium channel may be the temperature sensor and end with a model [89] developed thirteen years later where no single major sensor is proposed. This is a reflection of the probable reality that there are multiple thermometers in plants [72] and signalling pathways in plants that have complex interrelations [91]. Several sensing devices have been proposed to be upstream of the calcium response, [71] including membrane fluidity, protein conformation, cytoskeleton assembly status and enzymatic activities. But as Ruelland *et al*. [71] note, the relation between sensors and signalling, or upstream and downstream is not necessarily one-directional. For instance, possible temperature sensors such as membrane fluidity [92] may trigger calcium release and then be in turn reinforced by calcium signalling. Nevertheless, the search for the primary thermosensors continues, with recent studies on heat shock perception [86,93] indicating that cyclic nucleotide gated channels respond to changes in membrane fluidity.

## 5. Calcium and Symbiosis

**Figure 3:**
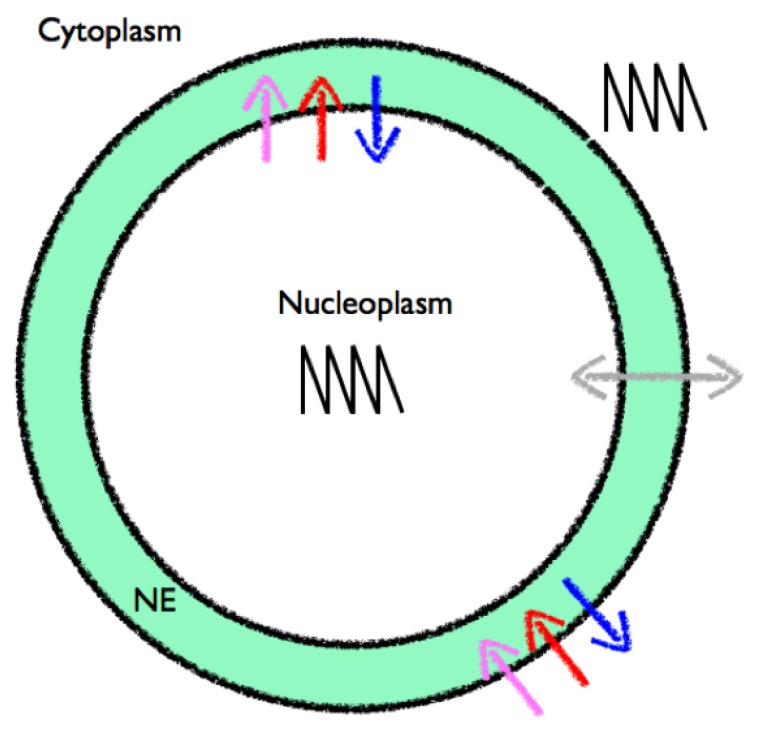
Schematic of the possible signalling components during symbiosis. Calcium can be released from the nuclear envelope (NE) into either the cytoplasm or the nucleoplasm via calcium release channels (blue). Calcium is actively removed from the volume back into the NE through the action of calcium pumps (red). Other transporters, such as a K^+^ channel, may be involved in voltage regulation (pink). Passage of calcium ions between the interior and exterior of the nucleus may be possible through the nuclear pores (grey). Spiking occurs within the nucleoplasm and in the nuclear associated cytoplasm. The communication between different sides of the NE is discussed in Section 3.3.

Exposure of legume root hair cells to rhizobial-derived nodulation (Nod) factors results in significant physiological and morphological changes that allow these bacteria to infect the plant in a controlled manner [42,94]. The rhizobia fix nitrogen for the plant within specially grown organs known as nodules and receive sugars from the plant in return. After detection of the Nod factors, but before gene expression, a cell-scale calcium transient followed later by sustained oscillations (known as Ca^2+^ spiking) in the nucleus and perinuclear space is observed (Figure 3) [95]. Mutants exist which are defective in the spiking response and also block symbiosis gene expression [24,96]. Furthermore, a mutant corresponding to a Ca^2+^ and Calmodulin-dependent kinase (CCaMK), also blocks gene induction but retains the spiking [97], and the activation of this kinase is both necessary and sufficient for the induction of nodulation gene expression [98]. Blocking Ca^2+^ channels and pumps chemically inhibits both the Ca^2+^ spiking [99] and gene expression [100]. All of this suggests that Ca^2+^ is essential for the regulation of nodulation. Interestingly, many of these components are shared with the symbiosis pathway between plant and *Arbuscular mychorrizae* fungi. CCaMK is able to induce the expression of different genes according to whether the plant detects Nod or Myc (Mychorrizal) factors [101].

Key questions in this field are therefore: (1) how are the oscillations established and what are the molecular mechanisms of the core components; (2) what is the role of the nuclear membrane in generating peri-nuclear and nuclear oscillations; (3) how are the calcium oscillations decoded to affect downstream developmental programmes; and (4) is there a role for calcium in determining the specificity of the Nod *vs*. Myc pathway? These final two questions are discussed in a more general setting in the specificity section towards the end of this review.

Different hypotheses have been put forward to explain the generation of perinuclear calcium oscillations. In Granqvist *et al*. [22] a three-component ODE system (Equation (1)) located on the inner nuclear membrane is presented containing a Ca^2+^-activated K^+^ channel [27], a voltage-gated Ca^2+^ channel and a Ca^2+^-ATPase [51] (Figure 3), described by equations similar to Equations (5) and (6), which in its simplest form is able to capture the observed global spiking behaviour. By including calcium-buffering species (e.g., proteins which bind calcium), the authors aimed to explain several experimental observations on the nature of the calcium signal that could not be accounted for within the buffer-free system. Firstly, that different spike shapes are observed when using different experimental techniques, secondly that the initial calcium spikes occur at a higher frequency than the later spikes, and finally that the oscillations terminate. Furthermore, imaging data is of calcium bound to a buffer and not the absolute value of the calcium concentration, so it is most useful to compare experimental data to Ca^2+^ bound to buffers within simulations.

By changing the buffer dissociation constant to known values [102] the model could reproduce the various observed spike shapes for the different buffers used experimentally. The period of high frequency spiking observed initially could be explained by the presence of large quantities of unbound buffer at the start. By hypothesising that the perception of the symbiont signalling molecule causes an increase in buffering capacity within the nucleus, possibly by the migration of calmodulin to the nucleoplasm from the cytosol as is observed in animal systems [103], the model predicted that a period of rapid oscillations would occur if additional quantities of the signalling molecule were added during existing oscillations. This was indeed observed, demonstrating that components beyond those identified in previous genetic studies play an essential role in determining the calcium signal.

A more unconventional approach to calcium signalling modelling was presented by Sciacca *et al*. [104] utilising a new methodology referred to as the Calculus of Wrapped Compartments (CWC) [105]. At its heart the CWC model is a term rewrite system [106], in which the biological system is described by a “term” and the evolution of the system is modelled by the application of a set of “rewrite rules”. The form of the terms and rules considered within the CWC allows the description of membrane wrapped compartments, and can simulate the interaction of elements localised to membranes and within the compartmental volumes. These elements can represent a diverse array of biologically important substances, from proteins to simple signalling ions, whose interactions are described through the use of the rewrite rules. An example term would be 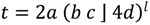. This describes a single compartment 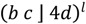, denoted *l*, with two elements, *b* and *c*, on its surface (perhaps representing membrane embedded proteins), four atoms of *d* within the compartment and two atoms of *α* outside the compartment (representing individual molecules, enzymes or other biologically relevant components within the bulk). An example rule would be

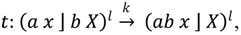
(7)
which represents the binding of molecule *b* by the membrane localised protein *α* to form the complex *αb* on the membrane, in the presence of any other membrane elements *x* and volume elements *X*. This reaction has a rate constant *k*, which determines the probability of this rule being applied to the term during the evolution of the system.

In describing nuclear-localised calcium signalling, Sciacca *et al*. [104] use a single membrane to describe the inner nuclear membrane and have calcium move between the nuclear envelope and the nucleoplasm by introducing rewrite rules that describe the release of calcium ions, denoted *Ca* in the rewrite rules, through calcium channels (*OCh* in the open state, *Ch* in the closed state) and the uptake of calcium back into the nuclear envelope by a Ca^2+^-ATPase,

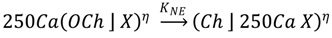
(8)

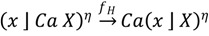
(9)


Channel opening results in an instantaneous nucleoplasmic Ca^2+^ increase at a 2.5% level of the Ca^2+^ in the nuclear envelope, while the calcium uptake is described by a Hill function rate (see Equation (6)). To cause channels to open, the authors consider the binding of an external signalling molecule, which transduces the signal from the symbiont to the nucleus,

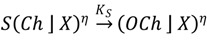
(10)


By considering this (as yet unidentified) signalling molecule, they show how a decaying quantity of the signalling molecule results in a calcium trace with decreasing spike frequency. Rather than attempt to directly compare the model-generated trace to the experimental traces, they focus on the information content of the spikes [107,108]. In this way, they show that the model captures the nuclear calcium dynamics in statistical terms.

Both Granqvist *et al.* [22] and Sciacca *et al*. [104] attempt to explain the observation of the variation in spiking frequencies observed experimentally: the initial spiking has a higher frequency than that observed later. Sciacca *et al*. [104] suggests that the Ca^2+^ channels are opened by binding some signal molecule, generated when receptors on the root hair cell exterior bind Myc factor. This signal molecule is degraded over time, resulting in a reduced likelihood of channel opening, thereby leading to a reduced spiking frequency. Granqvist *et al*. [22] instead explain the high frequency spiking by the initial presence of unbound buffers, that eventually saturate resulting in a stable, lower frequency, oscillation. They hypothesise that the binding of Nod factor results in an increase in buffer capacity within the nucleus. The direct interaction between Nod/Myc factor reception and channel gating may not be consistent with current biological models [27], however the CWC approach has great scope for assistance in explaining the symbiosis processes. The method is extremely versatile; within the same mathematical framework, the authors have been able to study the spatial interaction between the Mychorrizal hyphae and the plant root [109], a technique that could be extended to the interaction of rhizobia and the plant within infection thread growth/formation, for example. Within the sphere of calcium modelling, it is simple to add additional rules to incorporate different gating models (the calcium channel is yet to be identified and characterised experimentally) and buffering affects. Beyond modelling the symbiotic calcium signalling the approach could be valuable in describing multi-compartment interactions in whole cell signalling and intercellular processes.

Work on symbiosis signalling has so far concentrated on the temporal behaviour of the calcium signal. However, the spatial nature of the system can have important implications on the signals generated [63]. The signal within the nucleus is spatially inhomogeneous [60], and it is important to understand how the decoding protein perceives, and is affected by, the signal. Work studying the spatial dynamics of the symbiosis signalling [51] (described in Section 3.3) has so far been limited to the dynamics on the membrane.

## 6. Calcium and Polar Growth

The anisotropic cell expansion seen in polar, or tip, growth plays an important role in plant development. Root hairs and pollen tubes have both served as model systems for dissecting the mechanisms underlying polar growth. The role of modelling in elucidating the mechanisms underlying pollen tube growth has recently been reviewed in Kroeger and Geitmann [110,111]. Calcium has long been identified as playing a role in tip growth [112] as have oscillations in pH and ROS. Interestingly, both the Ca^2+^ concentration at the tip and the growth rate oscillate [113,114,115,116,117], suggesting a mechanistic connection between the two.

**Figure 4:**
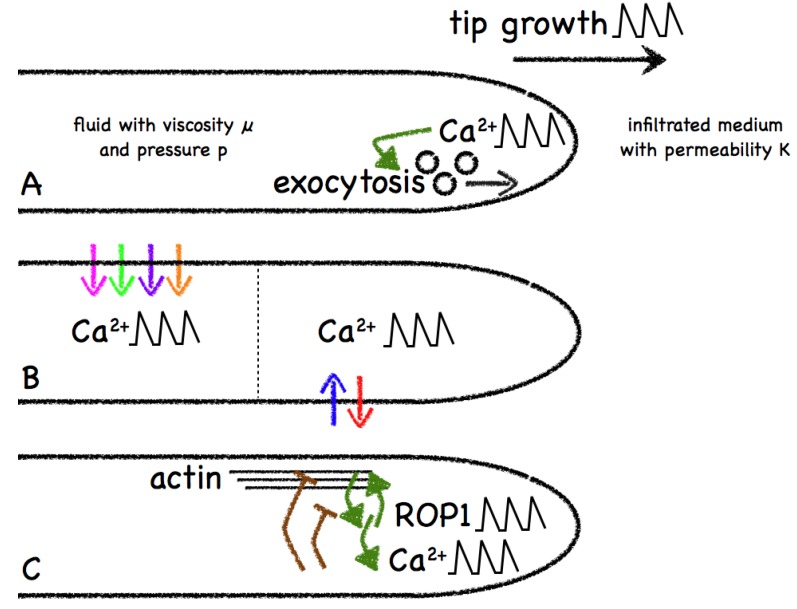
Models for the role of calcium in tip growth. Model **A** aims to capture the viscoelastics of tip growth by considering a fluid growing under pressure into another fluid. Calcium is hypothesized to have an effect on the exocytosis rate. Model **B** investigates the establishment of calcium gradients between compartments and the role of oscillations. Both the tip and shank region include transporters for the potassium, calcium, protons and chlorine. Gradients do not require oscillations for their establishment or maintenance. Model **C** studies the role of calcium on actin and ROP1. This model included a number of hypotheses that were evaluated both computationally and experimentally to put forward a validated network for the feedback between the various components of their model.

Kroeger *et al*. [118] developed a theoretical growth model to address the role of calcium in the observed oscillatory growth pattern of pollen tubes. Their work is of particular interest in that it links calcium patterns generated by a biochemical reaction diffusion mechanism to a dynamic biomechanical model for pollen tube growth. In order for cells to grow and maintain their structural integrity, wall expansion requires the addition of new wall material and the mechanical deformation of the wall (growing cell walls are typically about 0.1–0.5 µm thick). These processes are complementary and in order to maintain wall integrity it is likely they alternate during growth. Cell wall material is added by secretion and synthesis at the cell membrane and mechanical deformation of the wall is achieved as a consequence of stresses exerted by the internal turgor pressure of the cell, Figure 4A. Cell elongation was modelled as a viscous pressure driven flow with a stress-strain relationship and a continuity equation for pressure governing the process [119,120]. For incompressible fluids these relationships result in Darcy’s law,

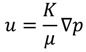
(11)
and a constraint for the pressure profile,

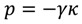
(12)
which together are known as the Taylor-Saffman relations. Here, *u* is the velocity of the viscous fluid, *p* is the pressure, *K* is the permeability of the infiltrated medium, *μ* is the viscosity of the injected fluid, *γ* the surface tension of the fluid interface and *k* is the interface curvature.

Experimental observations of a rigidity gradient due to methyl-esterification along the cell wall were described by an effective elastic constant of the cell wall. The elastic constant is the product of Young’s modulus and the cell wall thickness, both of which are time dependent. The elasticity of the cell wall is coupled to the calcium concentration at the cell wall; reduction of calcium softens the cell wall by preventing the gelation of pectin. In order to monitor the change of calcium concentrations over space and time, Kroeger *et al*. [118] used a reaction diffusion equation with a diffusion constant of 20 μm^2^/s in the cytosol and a diffusion constant of 0.03 μm^2^/s in the cell wall. Stretch-activated channels within the membranes are accounted for using the Einstein relationship for the diffusion constant. Using this relationship, the Taylor-Saffman equations and the conductance of a stretch-activated calcium channel, allowed the authors to estimate the number of active calcium channels at the tip and conclude there were approximately 10 at any given time. Cell wall thickness changes were modelled as being proportional to the cytosolic calcium concentration, following experimentally observed spatial correlations between high calcium concentrations and vesicle fusion rates. Kroeger *et al*. [118] predict that endocytosis acts as a stabilising factor for the oscillations. Endocytosis itself requires a calcium dependence within their model as a means of preventing the cell wall from becoming too rigid and to soften the cell wall when the growth rate is very small. They hypothesise that a role of endocytosis in tip growth is to remove calcium from the apical cell wall, thus preventing cell wall stiffening and allowing the cell to maintain sufficient plasticity for anisotropic growth. The model correctly captures the oscillatory behaviour of pollen tube growth, cell wall thickness and calcium concentrations. As expected from the inverse relationship between growth velocity and the effective elastic constant of the cell wall, the maxima of wall thickness and growth rate do not coincide—they oscillate with a phase delay relative to one another. Furthermore, the model predicts a phase delay between the cytosolic calcium concentration and the growth rate of about 150°. Such a delay is qualitatively consistent with experiments, but the quantitative value is at odds with the 30–40° observed by Holdaway and Hepler [121]. The authors then used their model to study the effect of external calcium concentrations and calcium influx on pollen tube growth rates. They found a linear relationship between the period of the oscillations and the external calcium concentration. In Kroeger *et al*. [122] this study was extended by replacing Darcy’s law with Lockhart’s equation [123] in order to account for wall stresses. Lockhart’s equation coupled with equations for cell wall rheology and calcium dynamics from their previous study [118] was used to evaluate the role of turgor changes in pollen tube growth. Using this approach, Kroeger *et al*. [122] found that calcium lags behind the growth rate with a phase difference of 50°, consistent with experimental values. This latest paper [122] thus nicely captures the core ideas of their earlier work [118] but adds a substantial refinement that results in a better description of pollen tube growth. Further experiments will be needed to test and validate the current model before refining it further. Next steps may include the extension to 3D and the investigation of different channel gating mechanisms.

Liu *et al*. [124] also studied pollen tube growth but their focus was on the maintenance of intracellular ion gradients and the relationship to oscillatory dynamics. Whereas at the tip, calcium, growth, *etc*. all oscillate, the pollen tube shank appears to be in steady state. In particular, the authors sought to address how stable cytosolic gradients are established, how the system deals with perturbations, and the role of oscillations in forming ion gradients. To address these points, a simple two-compartment model consisting of a tip region and the shank was constructed. The compartments are assumed to be homogenous and have the ability to exchange ions by diffusion and cytoplasmic streaming. Furthermore, both the tip and shank region include transporters for the potassium, calcium, protons and chlorine, Figure 4B. Guided by experimental observations they built a number of assumptions into their model: that calcium enters the pollen tube at the tip and there are different abundances of proton ATPases in the tip and shank region (accounted for by changing the parameters in the H^+^-ATPase pump). ODEs (Section 2.2) for the top and the shank are used to describe the temporal evolution of the ion concentrations in the model. This model can reproduce oscillations between 3.8 and 4.8 µM in the tip region, and also shows oscillations in the shank region but with an amplitude six orders of magnitude lower (7 × 10^−6^ µM), thus maintaining a “steady state” in the shank. Likewise the pH value oscillates at the tip but is stable in the shank. The authors find that oscillations at the tip are not important for establishing ion gradients, as would be expected from basic electrophysiological considerations.

This fixed compartment volume model is then extended using a power-law formalism (with all exponents set to 1) to account for growth as a function of the ion concentrations, thus forcing ion oscillations to lead to growth oscillations. Two further ordinary differential equations are added to account for volume changes at the tip and shank. To address the question of whether growth oscillations lead to ion oscillations, a sinusoidal growth rate was imposed. Two hypotheses are put forward from the model that could account for a feedback from growth onto the ion concentrations: firstly, the transition of tip membrane into shank membrane, and secondly a change in the transport kinetics as a function of growth. This work nicely demonstrates how simple models (in terms of spatio-temporal dynamics) can lead to some interesting findings that go beyond recapitulating experimental observations. It would be interesting to try to reduce this model further to distil out the Guided by experimental observations they built a number of assumptions core components in the system and then to build the model up to investigate the contribution from each new addition in more detail.

The models of Kroeger *et al.* [116,120] use stretch-activated channels, whereas the mechanism for calcium release in Liu *et al*. [124] is voltage-activated. In a model put forward by Yan *et al*. [125], increases in apical calcium influx are attributed to the ROP1 (RHO related GTPase) activation of the RIC3 pathway. The focus of this research was to understand the role of calcium in linking ROP1 and actin. One of the first models for the interaction between calcium and the cytoskeleton was put forward by Goodwin and Trainor [126]. They modeled the dynamics of calcium in the cortical cytoplasm of plant cells using mechanochemical field theory. Their model resulted in a system of two non-linear PDEs. This system was solved numerically [127] and investigated further in terms of oscillations by Briere, C. and B.C. Goodwin [128] and subjected to stability analysis [129]. The authors observe periodic, aperiodic and chaotic behavior in their models. This was perhaps the first successful attempt to take elements of the spatio-temporal organization of the cell as well diffusion and mechanical effects into account.

The mathematical model of Yan *et al*. [125] simulates the observed changes in ROP1 activity caused by F-actin disruption and suggests a role for calcium in the negative feedback regulation of the ROP1 activity, Figure 4C. The authors propose two different models to account for this feedback. The models predict that either calcium promotion of F-actin depolymerisation or calcium activation of ROP1 inactivators such as RopGAP is sufficient for the generation of oscillating ROP1 activity. They formulated the models as delayed differential equations with the delay characterizing the lag between ROP1 activity and calcium accumulation. The authors find that this delay is important for the establishment of oscillations and that a delay of 8 s was able to reproduce experimental observations with a period of 80 s. Both models are able to fit the data. They therefore investigated differences in the model behaviour for perturbations to the system, thereby suggesting experiments to validate their assumptions and predictions. They conclude that F-actin provides positive feedback regulation of ROP1 and that this feedback is key for oscillations. This assigns a critical role to apical calcium in the feedback for ROP1 activity and leads to predictions for elevated calcium concentrations that the authors followed up on by perturbing calcium levels. Consistent with their model they find that higher calcium levels lead to ROP1 depletion at the tip, whereas decreasing calcium maintains high ROP1 levels, but in both cases no ROP1 oscillations occur.

## 7. Calcium and Stomata

**Figure 5:**
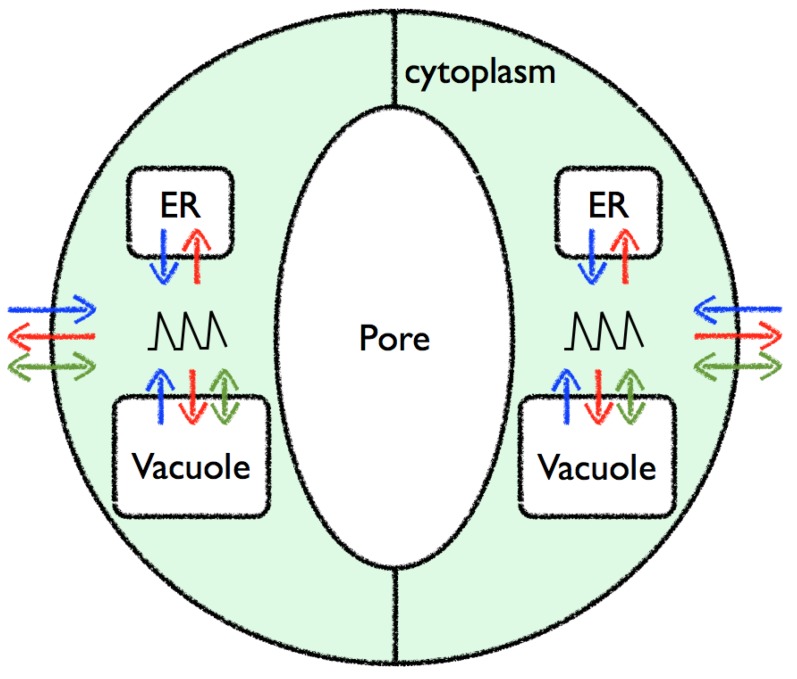
Sketch of a stomate showing a pair of guard cells containing the vacuole and the ER. Channels/pumps are indicated by the lines, with the arrows showing the ion transport direction. Exchangers are drawn with double arrows.

Stomata are microscopic pores found in the plant epidermis through which plants control gas exchange with their environment. The stomatal complex comprises the pore, a pair of guard cells that surround the pore and, in some species, subsidiary cells that separate the guard cells from the epidermal cells. A sketch of the stomatal complex is given in Figure 5 showing selected organelles and ion transporters. Stomatal opening and closing is the result of one or more biotic and/or abiotic factors, for example, CO_2_ concentration and the drought hormone, abscisic acid (ABA). These stimuli affect the plant, which in turn regulates the stomatal aperture by changing the guard cell volume by osmosis. When the volume of the guard cells increases the stomate opens, thereby increasing the gas flux into and out of the plant. The incoming CO_2_ is consumed by photosynthesis but at the expense of losing water vapour. The stomate closes when the guard cell volume decreases. The stomatal pore width therefore exerts a large influence on the rates of photosynthesis and transpiration.

The review of Kim *et al*. [130] and the references therein reveal that the signalling pathways in guard cells are complex and are mediated by several mechanisms including ion channel regulation. Ions, such as K^+^, Cl^−^ and Ca^2+^, play pivotal roles in these signalling networks. Modelling ion fluxes and their rates of changes leads to systems of differential equations of varying complexity as described in Section 2.2. Bayesian graphical models and Boolean networks [131] provide alternatives to ODE models, and are particularly useful at identifying an “optimal” network from a panoply of potential networks. *In silico* models of the networks and/or the ion flux regulation that leads to stomatal aperture changes can be used to complement wet-lab experiments, or possibly identify fruitful avenues for further study.

A Boolean-based network method was used by Li *et al.* [132] to investigate the signal transduction network of ABA, which causes stomatal closure and inhibits opening. The authors based their investigation on a small set of inference rules that merge two closely related processes, representing activation and/or inhibition, into a single process. Repeated application of the rules to the set of 121 experimentally identified processes yielded a signalling network incorporating enzymes, secondary messengers, signalling proteins and membrane transporters. In the network there were multiple redundant paths that link ABA to stomatal closure. Path analysis of the network does not capture cooperativity between signals and so the authors employed a dynamic model characterised by the on/off state of a node. Although this approach lacks the temporal dimension associated with reaction rates, it does provide insights when the individual processes have been identified but some or all of the quantitative kinetic parameters are unknown. To overcome the absence of kinetic data in the model the relative timing of each process and its initial state are chosen randomly. By an appropriate choice of the timestepping algorithm and a large number of simulations (10,000) the dynamic response was elucidated and the probability of stomatal closure was calculated and compared to the wild-type response. One or more nodes were then systematically switched off (thereby mimicking genetic knock-outs) to gauge the sensitivity of the network. The link between ABA and stomatal closure was completely severed in the model by the simultaneous disruption of four network nodes: actin reorganisation, cytosolic pH increase, malate breakdown and membrane depolarisation. Disruption of cytosolic Ca^2+^ increase predicts slower than wild-type closure, whereas a disruption to the Ca^2+^-ATPase node leads to a faster than wild-type response. To test the cytosolic pH predictions, the authors compared their results to experiments where the pH is clamped using the weak acid butyrate. This was expected to disrupt stomatal closure because cytosolic pH acts as a messenger during the process. Experimental results and predictions were consistent, *i.e*., increasing butyrate led to decreased ABA sensitivity. Further experiments were suggested based on the predicted network. The predictions suggest several novel interactions related to ABA responsiveness. In summary, the method provides a flexible framework which can be generalised and applied to other processes where quantitative information is absent or incomplete [133].

The network analysis of Li *et al*. [132] incorporated over 40 identified components in the ABA-signalling network of guard cells. The earlier theoretical work of Veresov *et al.* [134] examined the action of ABA but limited the scope to calcium signalling. Based on experimental observations, the authors proposed a model that included the endoplasmic inositol 1,4,5-triphosphate-sensitive (IP_3_-sensitive) channel and Ca^2+^-ATPase pump, together with the cyclic ADP-ribose-sensitive (cADPR-sensitive) channel and the Ca^2+^/H^+^ antiporter in the tonoplast. The reaction kinetics were modelled by a set of ODEs which were simplified by treating some of the differential equations as stationary. Kinetic parameters were obtained from the literature where available, e.g., cADPR-gated calcium release [135], and the remainder from simulations. The resulting model simulated oscillations in the cytoplasmic Ca^2+^ concentration for a range of ABA concentrations (0.01 and 1 µM) provided both channels were included. The model predictions showed good agreement with published experimental results for *Commelina communis* and for the two chosen ABA concentrations.

The increasingly extensive kinetic characterisation of the processes involved in guard cell aperture changes was brought together into the OnGuard model by Hills *et al.* [136]. The model integrated the cytosolic-free Ca^2+^ concentration together with pH, other ions, osmolite metabolism, membrane transporters, and a wealth of published kinetic and channel gating parameters into a system that represented a *Vicia faba* stomate.

The model considered the apoplast, the cytosol and the vacuole as three membrane-separated compartments where the apoplast was assumed to have infinite volume due to the use of experimental results from epidermal peels. The complexity of the cytosolic calcium buffering was dealt with by treating the process as a “black box” with a single calcium buffer. The model included a total of 25 membrane transporters: 11 on the plasma membrane and 14 on the tonoplast. Of these transporters, both membranes had Ca^2+^ channels and Ca^2+^-ATPase pumps, the tonoplast also had a CAX-type Ca^2+^/H^+^ antiporter and ten of the other channels were kinetically sensitive to Ca^2+^, e.g., the H^+^-ATPase on the plasma membrane. The ion flux through a channel was described as a function of several variables, including the membrane potential, channel open probability and the channel conductance, and the equation easily catered for voltage- and ligand-gated channel control mechanisms. The majority of the kinetic parameters for the model were taken from published experimental results for *Vicia* guard cells; however where values were unavailable published results from other species, e.g., *Arabidopsis*, were used. The stomatal aperture was introduced by equations that independently and linearly relate the aperture to turgor pressure and guard cell volume. A separate equation for the guard cell volume was derived in terms of the turgor pressure and concentration of osmotically active solutes. These equations provided a basis for an iterative method that linked the compartmental and membrane kinetics to guard cell volume changes and hence stomatal aperture.

The model was used to compute open and closed stomatal reference states given a set of solute concentrations and membrane voltages. The open state was found by setting the initial conditions to values typically found in an open stomata and the closed state was found by substantially reducing the currents of the primary pumps at the tonoplast and plasma membrane. These reference states provided configurations with which to test the parameter sensitivity of the model. The model was substantially less sensitive to changes in the transport activity parameters than to variations in the densities of transporters directly affected by unbound Ca^2+^ and pH in the cytosol. To mimic environmental scenarios, the model was interrogated as to the effects of changes to external solute concentrations. Published experimentally observed responses were captured by both reference states.

The absence of a diurnal cycle in the OnGuard model [130] was addressed in the follow-up work of Chen *et al*. [137]. The day-night transition was effected by introducing a hyperbolic dependence on light to the turnover rates of the primary membrane transporters (H^+^-ATPases, Ca^2+^-ATPases, H^+^-PPase) and the rates of sucrose and malate synthesis, with the latter two set to zero during the 12 h dark period. The 12 h light period was simulated by linearly increasing the light parameter from zero to a maximum and then back to zero. The diurnal cycle of membrane voltages, stomatal turgor, volume and aperture produced by the model, agreed well with observations. Predicted total vacuolar and cytosolic [Ca^2+^] showed the vacuolar concentration increasing during the day and the cytosolic concentration peaking sharply during the first few hours of darkness (as did the cytosolic-free [Ca^2+^]). The model predicted voltage and cytosolic-free [Ca^2+^] oscillations during the 3–4 h day to night transition that were noted to be broadly consistent with previous observations. The standout prediction, however, is the diurnal variation in the resting cytosolic-free [Ca^2+^], in particular the significant daytime increase despite the enhanced export of Ca^2+^ from the cytosol during this time. This counterintuitive prediction was explained as a result of plasma membrane hyperpolarisation, which limits the kinetics of the Ca^2+^-ATPase and Ca^2+^ channels located there. This mechanism provided an explanation without the requirement for a feedback loop, as suggested by Dodd *et al*. [138], although the authors point out that one does not preclude the other. It should also be noted that the investigation of Dodd *et al*. [138] was experimental and only in Arabidopsis whereas OnGuard [130] is kinetically characterised by multiple species.

Using a variety of methods the authors of the works described above are able to prise out novel predictions. The work of Li *et al.* [132] utilises a Boolean-network approach to synthesise the ABA signalling network from a set of known process and then successfully tests one of the predictions experimentally. This approach however is criticised by Chen *et al*. [137] due to the availability of kinetic parameters, albeit drawn from several species and cell types. By using a kinetics-based model Veresov *et al*. [134] were able to quantitatively predict experimentally observed calcium oscillations. The results of Veresov *et al*. [134] are included in the review by McAinsh and Pittman [139] who provide a list of the Ca^2+^ transport pathways and also refer to the work of Li *et al*. [132]. The OnGuard system of Hills *et al*. [136] brings together the increasingly comprehensive knowledge of the guard cell and generates reference states that accurately represent an open and a closed stomate. Chen *et al*. [137] introduce a diurnal cycle to the OnGuard system to model the effect of the day-night cycle on the stomate. Their work not only successfully captures experimental observations but also facilitates novel predictions.

## 8. Calcium and the Circadian Clock

The diurnal cycle has a significant effect on calcium behaviour [137,140]. Living systems internally measure and react to the diurnal cycle by means of the circadian clock, a genetic network that regulates the rhythms in biological processes throughout the organism. The unravelling of gene regulatory networks and mechanisms underlying the circadian clock in plants has benefitted significantly from contributions from mathematical modelling [141,142]. A main goal of this research has been focused on understanding the entraining and robustness of these networks and has led to some important insights as well as new genes and suggested further experiments [143]. That calcium base levels oscillate with a circadian period had been reported but until recently the feedback mechanisms had not been elaborated on. Not only the circadian oscillator but also light signalling is known to influence oscillations in cytosolic free calcium concentration and the phase of this oscillation changes in response to photoperiod. Using reverse engineering and control theory, Dalchau *et al*. [144] investigated whether this dual regulation might be determining the phase of the oscillations to test the external coincidence hypothesis, which states that the resulting phase arises from the coincidence between the phase of the oscillator and that of the external light and dark cycle.

The issue of missing or poorly defined parameters is well known to most systems biologists, as are ways of dealing with this. It is common to proceed with parameters from other experiments, often with different organisms under different conditions, such as in the OnGuard model [136] described above, or to optimize the parameters to fit the available data and then use further experiments as validation to iteratively improve the model and its parameters. Other approaches include simplifying the description of the system to find an analogous description that is still adequate but that requires fewer parameters, such as approximating mass-action kinetics by Boolean networks. These approaches are still typically based on what we think the underlying gene network is or approximations thereof. Given the amount of data and the nonlinearities of the models, parameter inference can quickly become a computationally daunting task. This is even more pronounced for methods that attempt to take the underdetermined nature and uncertainty of the parameters into account, such as Bayesian inference and Markov Chain Monte Carlo variants.

Another interesting way of tackling the issue of unknown parameters is the approach used most successfully by Dalchau *et al*. [144] in which the authors essentially move from the exploration of parameter space for a given model to sampling a model space of linear time-invariant models with delay parameters (the delays correspond to interactions between the inputs). Rather than aiming at fine-tuning a mechanistic model to existing data, this approach (known as systems identification or genetic programming) seeks to describe the available data with a small set of linear differential equations. The advantages of using small linear systems are massive in terms of the ease of parameter estimation, allowing large sets of models to be evaluated rapidly. This combined with model validation with data unseen by the fitting process allows for the model space to be explored and good models selected. Dalchau *et al*. [144] used this approach to derive mathematical models from bioluminescence data for oscillating cytosolic calcium with light and the promoter activity of the clock control gene, CCA1, as input.

By using so-called hidden variables in the model it is possible to account for further complexities in the connections between variables, *i.e*., to unknown, uncharacterized or non-modelled pathways. Hidden variables arise in the model when the number of measured outputs is less than the number of equations describing the system, *i.e*., some of the outputs are not observed. Adding hidden variables gives more complexity to the system but this needs to be justified by increasing good fits to the data. Information-based criteria exist for guiding this model extension process and reducing the risk of over-fitting [145,146]. Optimal models were found to have one hidden variable in this example. By simulating effects of mutations in CCA1 and the hidden variable and comparing these observations from CCA1 and photoreceptor mutations, the authors were able to draw inferences regarding possible biological meanings of the hidden variable. The model demonstrated that oscillations were controlled by both rapid light signals and circadian inputs. This results in an incoherent feedforward loop for the control of cytosolic [Ca^2+^] by light. The relative importance of both nodes in this feedforward loop was estimated by frequency domain analysis (Bode plots). The hypothesis that rapid light input is associated with correct circadian timing was tested by subjecting over 3.5 thousand rhythmic transcripts from published microarray data to a Bode analysis. Over one thousand of these resulted in good models. The authors suggest that for the remaining transcripts their linear systems are not complex enough to capture the observed dynamic behavior.

Dalchau *et al*. [144] thus took an interesting approach in this work by using a control theory framework that may be of wider use in modeling calcium dependent processes with sparse data. A valuable lesson can be learnt from the success of this approach. Whilst using low-order linear equations to describe biological systems clearly departs from our understanding of what is taking place mechanistically, the approach was nevertheless a sensible approximation given the amount of data available [147]. Modeling systems at the mechanistic level is often desirable as the parameters of the model often map directly onto biological quantities, such as concentrations or binding constants that can be perturbed. However, given the typical amount of data to fit the models, the parameters are often very poorly defined and/or multiple consistent solutions exist. The idea of focusing on predictions rather than parameters [148] could therefore be extended to focus on predictions rather than models or parameters. Indeed, within the Bayesian framework if these are uncertain they should be integrated out. It would be interesting to contrast this exploration of linear time invariant systems with an analogous approach based on sampling function space during regression as is employed in Gaussian Processes [149] and Bayesian approaches for handling parameter uncertainty to understand better the constraints on model and parameter space imposed by typical biological data.

## 9. Calcium and Systemic Responses

**Figure 6:**
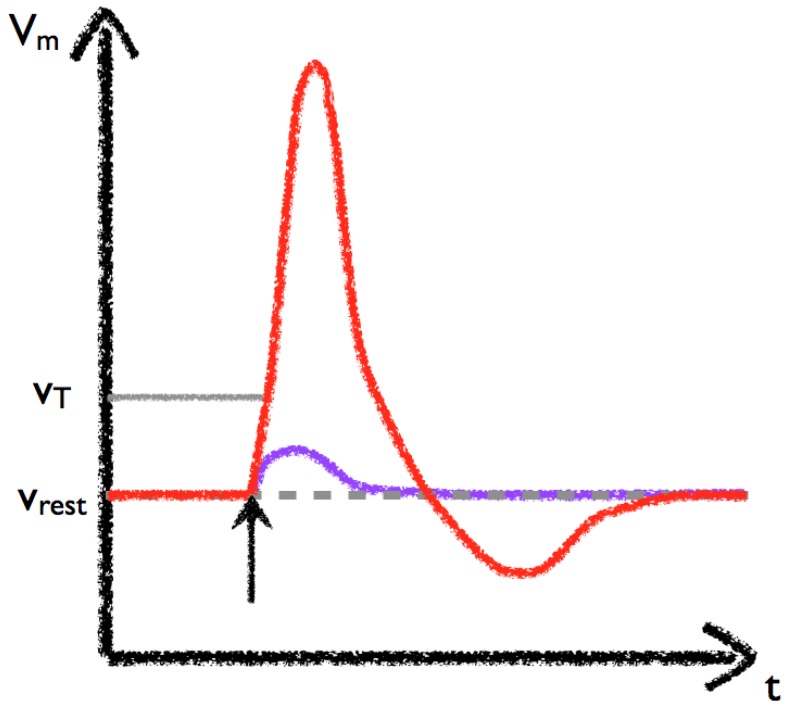
Schematic of an action potential, showing the variation of the membrane voltage, V_m_, at a single location as a function of time. A stimulus of various magnitudes applied at the time indicated by the arrow triggers a response that is dependent on the magnitude of the stimulus. A small stimulus (purple) results in a small membrane depolarisation that rapidly returns to the resting potential (v_rest_, dashed). A large stimulus (red) is sufficient to trigger Cl^−^ channels that result in a large membrane depolarisation (the action potential). The initial depolarising stimulus must be greater than some threshold value, v_T_, (indicated by the grey line) to trigger the Cl^−^ channels and cause the development of the action potential. The action potential is an all or nothing response, any supra-threshold stimuli will result in the same action potential response.

Electropotentials are generated in response to a range of biotic and abiotic stimuli and are a means of rapid communication eliciting responses far from the original stimulus. The two main forms that have been modelled are action potentials (APs) and variation potentials (VPs) [150]. The generation of an electropotential is associated with the passive fluxes of the ionic species Ca^2+^, Cl^−^ and K^+^ [150,151]. An initial influx of Ca^2+^ triggers a Cl^−^ efflux through voltage-dependent anion channels, which results in a rapid depolarisation of the membrane (see Figure 6), which is known as an electropotential. This depolarisation further activates outward K^+^ channels that are responsible for a slow repolarisation phase. APs and VPs are responses to different stimuli. An action potential is triggered by a non-damaging stimulus (e.g., cold, pH changes, salt stress), and is propagated by voltage-dependent Ca^2+^ channels, whereas a variation potential is generated in response to damaging stimuli (e.g., wounding, chewing insects) and is propagated by mechanosensitive [152] or ligand-gated [153] Ca^2+^ channels.

Sukhov *et al*. [154] proposed a model for AP development. The model incorporates the transport of ionic species (H^+^, Ca^2+^, Cl^−^ and K^+^) across the plasmalemma, including five carriers (inward and outward K^+^ channels, Cl^−^ channels, H^+^-ATPase, 2H^+^/Cl^−^ symporter) as well as cytoplasmic and apoplastic buffers on the basis of the model by Gradmann *et al*. [155,156]. Additionally the model includes a H^+^/K^+^ antiporter, a Ca^2+^ channel and a Ca^2+^-ATPase. The aim of the model was to demonstrate the important influence of H^+^ on the generation of APs. They suggest that the Ca^2+^ influx inhibits the export of H^+^ at the same time as activating the Cl^−^ channels. Initially, changes of the membrane potential due to electrical stimulation and gradual cooling are modelled and compared to experimental data to validate their model. By decreasing the activity of the H^+^-ATPase, the authors show that the amplitude of the AP is reduced, and if the portion of inactivated pumps is in excess of 50% the AP does not develop at all, demonstrating that AP development requires H^+^-ATPase activity. They further show that Ca^2+^ interacts with the H^+^-ATPase as suggested, but that this interaction is not required for AP development.

In Sukhov *et al*. [157], the authors extend their previous work to study the propagation of APs through a plant tissue, presenting the first detailed model of AP propagation in plants. Cells from the previous paper were connected together to form a grid, allowing electrical conductivity and diffusion of apoplast ions between nearest neighbours. The development of an AP is a slow process in plants so the Hodgkin-Huxley equation for the membrane potential is replaced by a stationary one [158,159]. The difference between the current and the stationary membrane potential during simulations is assumed to be small for the conductivities used, which results in significant computational savings. The model is able to capture the quantitative details of experimental AP propagation when examining behaviour of the membrane potential. Sukhov *et al*. [157] go on to test the effects of cell-to-cell conductivity and the activity of the H^+^-ATPase on signal propagation, finding that increasing cell-to-cell conductivity and decreasing the membrane potential threshold (corresponding to decreased activity of the H^+^-ATPase) accelerates AP propagation. Interestingly, it is seen that properties that result in good AP propagation are generally bad for AP generation.

The only VP model to date was presented in Sukhov *et al*. [160], which extended the authors’ AP work. The authors consider a ligand-gated Ca^2+^ channel that is triggered by the binding of some “wounding substance” that diffuses from the wounding site through the plant xylem. The model was able to simulate VP propagation and showed qualitative agreement with experiments. The decaying ligand concentration away from the site within the model results in a decreasing VP as has been observed [161]. They demonstrate that the fluxes of H^+^ and Cl^−^ have an important effect on the characteristics of the VP; H^+^ inactivation leads to a longer lasting depolarisation phase, while the VP impulses are caused by Cl^−^ channel activation. However it was difficult to characterise these effects independently of the Ca^2+^ channel behaviour.

The models of AP generation and propagation by Sukhov *et al*. [154,157,162] focus their investigations on the proposed inhibition of a H^+^-ATPase by the Ca^2+^ influx. Conceptually we might expect reducing the efflux of positively charged ions from the cell would be beneficial to creating the membrane depolarisation seen in an AP, and reactivation of the pumps would aid in repolarisation, as is observed. The result that complete inhibition of the H^+^ pumps for the duration of the AP reduces the amplitude (or kills generation altogether) is unexpected, showing that the development of an AP is a more complex process than previously thought.

The behaviour of the H^+^-ATPase, along with the cell-to-cell conductivity, was seen to have an important effect on propagation, due to its influence on the membrane potential threshold for propagation. Sukhov *et al*. [157] demonstrated that those cells that are optimised for AP propagation, such as phloem sieve elements, struggle to generate APs. This explains the observation [150] that AP generation is likely to occur elsewhere and move into the phloem, where it can be propagated to reach the signal target. In all their models (AP and VP), the authors neglect other Ca^2+^ sources that may be important, such as vacuoles or the endoplasmic reticulum. It was shown [163] that internal stores were the main source of Ca^2+^ release during AP generation. Furthermore, both the tonoplast and plasmalemma membranes undergo excitation, suggesting that these other ionic stores could have a significant impact on generation of plant APs.

## 10. Calcium and Specificity

The specificity question is recurrent in the field of calcium signalling research: how can the same simple messenger (that isn’t chemically altered) convey information about the stimuli to elicit an appropriate response? Notwithstanding other possible elements acting in parallel along the signalling pathway, an appealing hypothesis is that the spatio-temporal properties of the Ca^2+^ signal encode information about the particular stimulus and determine the specific elicited response [139,164]. Whereas in animal systems this hypothesis has found wide experimental support, experimental evidence remains scarce in plants, and it is still not known if changes in calcium ion concentrations in plants act as more than just essential chemical switches [165,166]. Modelling can uncover the factors behind a particular calcium signature, distinguishing those that depend on the stimuli from those that are cell-specific. On the decoding side, it can also suggest biological reasons for a given signature. A key prerequisite of untangling the specificity question is the knowledge of the signal.

### 10.1. Inferring Calcium Signatures from Calcium Time Series

High noise levels, cell movements and experimental artefacts such as fluorescence bleaching hinder the accurate detection of the characteristics of the Ca^2+^ signal. Typical methods such as Fourier analysis or wavelets typically struggle with short noisy signals. To reduce these limitations preprocessing techniques are often employed. However, the applied data transformations may lead to the loss of relevant information. Granqvist *et al*. [167,168] use Bayesian inference [169] and express data uncertainties in terms of probabilities. Bayesian spectral analysis [170] begins by the formulation of a model of the observed data parameterised by the angular frequency and the amplitude. A prior distribution is assigned to the amplitude, and by integrating over it and using Bayes’ rule, the most probable frequency can be inferred by analysing the full posterior distribution. Nested sampling [171] is used for selection between alternative models, being especially useful in the presence of multiple frequencies. The method is applied to calcium spiking observed in *Medicago truncatula* root cells during plant-microbe interactions.

Even acknowledging the many uncontrollable factors that perturb the gathering of calcium data, it is important not to assume that random effects are necessarily behind any less regular appearance of calcium time series. Deterministic chaos can easily be mistaken for noise [172], as chaotic systems are highly sensitive to initial conditions. Kosuta *et al*. [173] resort to a variety of mathematical methods—tests for determinism [174], indicators of nonlinearity and the calculation of Lyapunov exponents [175]—to distinguish noise from chaos in cells undergoing either Myc- and Nod- factor-induced calcium oscillations. Showing that in most cases the exponents are positive for both symbiosis, they suggest that calcium spiking may be chaotic [176]. The chaotic nature of these signals may have implications for specificity. Interestingly, Buschmann *et al*. [25] note that the influence of an independent calcium oscillator on voltage-gated membrane transport can create irregular (even chaotic) behaviour of membrane voltage, and the model of Granqvist *et al.* [22,167] has a chaotic regime, although not in biologically relevant parts of parameter space. Given the discrete nature of individual signalling components, we can expect that for low concentrations, the ODE based approach will cease to become a good approximation and stochastic simulation methods will become better suited. How such seemingly random discrete events are coordinated to produce global scale oscillations and waves remains a challenging question at the heart of calcium signalling. The use of new calcium reporters, higher resolution microscopy and advanced modelling techniques are bringing us closer to addressing such problems and determining what signal the decoding-proteins are challenged with.

### 10.2. Different Stimuli Alter the Functioning of Transporters and Buffers

The calcium signature is primarily shaped by the influx and efflux systems. If stimuli alter the functioning of the transporters then information about stimuli may be encoded in the calcium signature.

In Bauer *et al*. [18] the information about the stimuli is conveyed by a Ca^2+^ oscillation frequency that increases with the Sr^+^ dose that opens the calcium-permeable channels, at the same time that the amplitude decreases. Likewise, Brière *et al*. [49] used the fact that different stimuli are associated with different shapes of calcium transients, to guess how variations in pH or temperature may affect the key processes of the model, such as the influx rate or buffer’s binding properties.

Bose *et al*. [83] adapt the model of Bauer *et al*. [18,23] to capture the influence on the cytosolic Ca^2+^ signature of two kinds of efflux systems: ATPase pumps with high affinity but low capacity, and CAX (calcium exchangers) with low affinity but high capacity. They show that varying activities of ATPase and Ca^2+^/H^+^ exchangers generate different Ca^2+^ cytosolic signatures on a short time scale. While the exchanger merely shifts the location of the first Ca^2+^ peak, the ATPase pump changes in addition the speed with which the Ca^2+^ concentration drops. Like Brière *et al*. [49], they fit the model to experimental data of responses to various stimuli. Using realistic initial conditions, they demonstrate that the model can adequately reproduce the response to cold, osmotic stress, touch and H_2_O_2_ treatments by changing the proportions of ATPases and exchangers. Most importantly, the modelling results are in agreement with the expected physiological changes under stress.

This indirect influence of the stimuli on Ca^2+^ transients shows that the calcium signature is mediated by the particular conditions of the system. As long as it is possible to experimentally measure those conditions, it should be possible to infer the shape of the calcium signature induced by changing levels of specific stimuli. But regardless of how they are stimulated, different cells can have different types of transporters. Presumably this would imply that the same stimuli might induce different calcium patterns. Moreover, cells can also differ within the same system, raising questions about the meaning of the calcium signature when calcium levels are measured at different scales or at different points in the system. In particular, Bose *et al*. [83] suggest that the experimentally observed presence of a second peak after an anoxia treatment may result from the heterogeneous oxygen profiles of the cells that exist even in the unstimulated plant, which are unlikely to coincide with the presence of different decoding elements. It would be interesting to know the extent to which the results of this model can explain previous observations in other contexts: for instance, Kiegle *et al*. [177] investigate cells belonging to different tissues exposed to various stresses and report distinct signatures.

### 10.3. The Experimentally Recorded Signature May Be Very Different from the Relevant One

Plant calcium signalling occurs within and between living, dynamic plant cells, often resulting in noisy single-cell data [178]. The reported calcium signature is, therefore, commonly based on measurements averaged over several cells. However, this averaging may miss subtleties and differences in the local patterns that decoding proteins are challenged with. It is important to know how a local specific calcium response propagates over a long distance, and how much information about the local response is retained at the global level. To begin to address these questions, Bose *et al.* [83] average the calcium kinetics over a population of many cells with a normal distribution of the pumps and exchangers, showing as expected that the integrated calcium signature has a lower amplitude and broader oscillations than the individual cell. A similar comparison was conducted by Dodd *et al*. [140] who used low-temperature-induced Ca^2+^ signals to investigate whether the circadian clock modulates Ca^2+^-based signalling pathways. Low temperatures induced transient increases in cytosolic [Ca^2+^] throughout the plant and they preceded stomatal closure. The authors noted a difference between repetitive Ca^2+^ oscillations seen in individual guard cells and the “spike-shoulder” pattern observed for a population of guard cells. They investigated whether low-temperature-induced [Ca^2+^]_cyt_ increases measured from a population of guard cells were likely to represent the summation of cold-induced single-cell [Ca^2+^]_cyt_ oscillations. In their model, the single-cell cytosolic Ca^2+^ concentration was represented by a modified sine-wave with peaks and troughs that decayed with time, and with the period treated as a normally-distributed random variable (mean 154s [179]). The authors show that the summation of a sufficient number of individual oscillations could produce the observed population level signature. The result implies that the difference between the calcium kinetics measured at the level of an individual cell and of many cells may not be an experimental artefact. Moreover, it implies that the information of the global signature may be misleading if we want to investigate the stimulus-specific calcium code that is relevant at the single-cell level.

To study in more detail the mechanisms underlying the relation between the local and global signatures, Plieth [180] proposes to model the propagation of Ca^2+^ signals between cells. The excitation of a cell results in a calcium spike described by a sum of exponentials, and can be propagated to neighbour cells according to cellular automata type rules. Not surprisingly, the overall signal mostly differs from the individual cellular response when few cells are excited at the start, or there are many cells, or when it is difficult to transmit the excitation because of high thresholds for excitation. Most interestingly, this modelling approach permits the consideration of a great variety of complex geometries common in plants. The definition of the neighbours depends on the shape of the plant; certain geometries will lead to a successive stimulation of a cell at a time whereas others will lead to the simultaneous excitation of several neighbours at once. The architecture of delays determines how much information about the individual signal can be transmitted to the whole plant. To demonstrate, the author shows that when the morphology is elongated, branched or dendritic the shape of an individual spike is not recognisable at a global level.

Moreover, in those more complex shapes, the capacity of the first simulated cell to transmit its signature to the entire plant depends on its particular location on the plant. If this is true, we can conclude that the shape of the cell organelles influences the calcium signature. Therefore care must be taken during experimental measurements to avoid inferring from the global signature the local signal that a decoding protein is confronted with.

Cellular automata type models commonly refer to binary behaviours. In Plieth’s model [180] a cell is either excited or not, and all the individual excitations look the same. Going beyond a binary rule could help clarify how the different geometries may affect the transmission of the specific calcium kinetics from the place where the plant was stimulated to a distant cell. This is relevant for instance during symbiosis, where it was shown [43] that particular Ca^2+^ spiking responses are associated with different key stages of transcellular infection. Moreover, the efficacy of cell-to-cell signal transmission must necessarily depend on the cell compartment that initiates calcium signalling. In a symbiosis context, Ehrhardt *et al*. [181] observe that adjacent cells have an autonomous response to rhizobium nodulation signals; arguably, we expect that if calcium spiking originates in the nucleus [47] propagation to adjacent cells should be less efficient than if it originated near intercellular membranes.

### 10.4. Finite Oscillatory Signals Are Optimally Decoded by Specific Proteins

Ca^2+^ elevations are finite in time and if they last for long they tend to be oscillatory, with the interplay between channels, Ca^2+^ feedback and pumps giving rise to periodically changing calcium levels. Beyond the biological advantage of reducing the time of exposure to toxic calcium levels, the specificity hypothesis implies that finite oscillatory signals encode information. Marhl *et al.* [182] focuses on the decoding side, discovering a reason for the finite duration and oscillatory nature of the signals.

Assuming that an optimal response is equivalent to maximal steady-state activation it is puzzling how this steady-state can result from a non-stationary signal. Most surprisingly, the termination of calcium signals also seems to contribute to an optimal response: while at least 36 [183] consecutive Ca^2+^ spikes are needed to trigger Nod-factor-dependent gene expression in *M. truncatula* root hairs, in the guard cells of *Vicia faba*, a train of exactly 5 spikes is able to achieve a half-maximal stomatal aperture [184,185]. This raises the question of whether and why the finiteness of calcium transients is instrumental in the decoding process. To answer this question, the authors simulate a train of 5 squared-shaped pulses to investigate the maximal steady response that distinct proteins with various kinetics rates of calcium binding and dissociation can reach. They find that if the kinetics of association and dissociation is fast, the activation of proteins will decay and rise following the oscillatory calcium signal, never reaching a stationary output signal. On the other hand, proteins with slow kinetics cannot reach a maximal steady state for the duration of the calcium signal. The definition of slow and fast kinetics only makes sense in relation to the frequency of the calcium signal: for each frequency there exists a protein with optimal kinetics, not too fast that it oscillates with the signal and not too slow that it cannot reach a maximal steady state. 

Response specificity will depend on the affinity of the different sensors for the particular Ca^2+^ signal, resulting in a further positive biological reason for oscillatory signals beyond the toxicity argument, as constant signals cannot activate proteins in a resonant-like manner. Naturally, if the calcium signal lasts for a long time, even slow proteins can become maximally activated over time. The finiteness of the signal is essential for the selective activation of the proteins, an effect that the authors name Finiteness Resonance.

An autonomous signal waiting to be decoded by proteins that bind to calcium underlies the analysis of Marhl *et al.* [182], but there are reasons to question this static view. Proteins are in general predominantly either buffers or sensors, but sometimes their roles are blurred [186,187] and in high concentrations any sensor acts as a buffer. In the model of Marhl *et al.* [182], in principle any binding protein could be considered as a buffer, and thus modulate the spatio-temporal characteristics of the calcium signal. Granqvist *et al.* [22] showed how buffers can vary the frequency of the signal and be instrumental for its finite duration. If the decoding proteins modify the calcium code, this complicates the decoding process and the interpretation of the experimentally measured calcium signature. However, not all buffers can act as sensors, and it should be possible to terminate a signal by injecting buffers in different amounts to investigate the resonant effects of signals with different durations. Higher resolution imaging of local Ca^2+^ fluctuations coupled with models that account for the feedback of calcium binding back onto the calcium signal will be required to unravel these finer details of the decoding process.

## 11. Conclusions: Calcium and the Experimental-Modelling Cycle—Past, Present and Future

We have presented a current snapshot of how mathematical modelling is helping to elucidate mechanisms and provide insights into calcium signalling in plants. A number of selected examples demonstrate the value of this approach, that when combined into a modelling-experimental cycle, can greatly enhance progress towards understanding the physical basis of signal generation, signal transmission and signal decoding. Mathematical modelling is thus contributing to unravelling the mechanisms of calcium signalling in plants and bringing us closer to a quantitative understanding of such processes. The models are summarised in Table S1 and some useful software given in Table S2.

This is a hugely exciting time for plant signalling and we are witnessing great leaps in progress in a number of areas, thereby moving some of the grand challenges we face in agriculture and food security within our reach. Calcium signalling plays a key role in many of these challenges. A number of important questions remain open, such as understanding the mechanistic link between calcium, ROS and systemic signalling, the crosstalk between calcium and plant hormones such as auxin or cytokinin, the influence of calcium on biomechanics, a detailed reconstruction of the local calcium signatures during various processes, mechanistic insights of specificity and the regulatory role of calcium in plants, a molecular level understanding of signal decoding, to name a few. We therefore anticipate an increasing role for systems biology and mathematical modelling to drill down towards the physics of these processes.

The power of modelling in this area is, however, very much dependent on equivalent advances in data collection and, in particular, imaging. The field has benefitted massively from breakthroughs in visualising calcium ions within live cells. For instance, Gilroy *et al.* [188] employed a fluorescent indicator to visualise and quantify the Ca^2+^ concentration in protoplasts, reporting a basal concentration of 171 nM and leading the way for many similar quantitative studies. Miller and Sanders [189] developed a Ca^2+^ selective intracellular microelectrode to measure basal Ca^2+^ as a function of light (from 150 nM to 400 nM). Also using Ca^2+^ selective microelectrodes, Felle [190] showed that auxin induces Ca^2+^ oscillations from 119 nM to 300 nM in maize coleoptiles. Another example was the induction of stomatal closure by ABA, which was accompanied by an increase in [Ca^2+^]_i_ to 600 nM in *Commelina* guard cells that had been injected with the fluorescent indicator dye fura-2 [191]. Of special mention is the work of Knight *et al*. [68] who introduced aequorin into tobacco plants and were able to show that a variety of stimuli induced Ca^2+^ stimulated luminescence. For further examples from a historical perpsective see Hepler [192].

Fluorescent proteins (FPs) and dyes are now prevalent in the study of calcium signals in cells [193]. The fundamental property that dyes and FPs exploit is the emission or alteration of their visible light spectra upon calcium binding. The techniques required to accurately infer the calcium concentration using the signals obtained from the chosen indicator are covered comprehensively elsewhere (for example see Fricker *et al*. [194]). The isolation of green fluorescent protein (GFP) in the 1960s led to its widespread use and the discovery of many other FPs, e.g., RFP, YFP, CFP and yellow-chameleon (YC). The use of genetically encoded calcium indicators (GECIs) is now widespread [195].

Apart from the diverse set of GECIs there are many synthetic dyes, e.g., fura-2 and indo-1, which report altered emission spectra upon calcium binding and are therefore used to infer the calcium concentration. Although the means by which GECIs and dyes are introduced to cells are different (GECIs are transfected and dyes are loaded) they are both measured using fluorescence microscopy. By accurate calibration of the signal-to-Ca^2+^ ratio the signal is used to infer an intracellular high-resolution Ca^2+^ map for the region of interest. The use of indicators suitable for ratiometric analysis is generally preferred due to their ability to avoid signal distortion due to effects such as heterogeneous indicator distribution. With respect to indicator choice, better resolution may be achieved by a prudent choice between similar indicators, for example YC3.6 and YC4.6 are both ratiometric indicators with the latter more appropriate in situations with a higher background Ca^2+^ [196]. Further diversity to the YC group has been achieved with the advent of YC-nano indicators [197] each with distinct emission signals at nanomolar [Ca^2+^]. It will be interesting to see whether the snapshot reporter concept proposed by Tsien [198] for perineuronal net imaging might be transferable to plants. The idea hinges on the simultaneous expression of reporter and effector proteins at a specific [Ca^2+^] when an external trigger (light is suggested [198]) is applied. The result would be a retrospective Ca^2+^ map with high spatio-temporal resolution. The development of calcium reporters with enhanced properties suited to the system under study and question are likely to facilitate an improved imaging quality that, coupled with microscopy developments, may provide new insights into the role of calcium in plant signalling.

The quantification of *in vivo* observations is likely to transform theoretical biology; a major limiting issue faced by modellers is the availability of experimentally quantified parameters for use in their models. The development of experimental processes that can help fill such gaps will be of huge benefit to the development of our understanding and will result in more accurate models. One such experimental technique that may be of great benefit to the field of calcium signalling is Fluorescence Correlation Spectroscopy (FCS) ([199,200,201], reviews: [202,203]). FCS involves a statistical analysis of intensity fluctuations arising from number fluctuations from, for example, molecules diffusing into and out of a small detection volume. Correlation analysis of the resulting signal can be used to extract local concentrations, diffusion coefficients, binding parameters and structural dynamics of important biomolecules *in vitro* as well as *in vivo*. It has been used, for example, to study the diffusive properties of the Ca^2+^ binding protein calmodulin [204]. The technique can be used with a standard confocal microscope, and advances in such technology have allowed the application of FCS to entire image stacks, in which each pixel becomes a detection volume. This enables the construction of diffusion or flow maps for the fluorescently tagged particles [205,206]. Consideration of the cross-correlation between different cellular compartments allows for resolution of diffusion barriers, as has been applied to the study of single molecule translocations through nuclear pores [207]. With sufficient temporal resolution, such an approach could be of great use in determining fluxes of calcium through channels during signalling processes.

A single fluorescent molecule may be about 1–2 nm in size but when imaged through a microscope this nanoscale source of light appears as a fuzzy spot with an intensity profile that defines the point spread function (PSF). If the distance that separates two identical sources of light is less than the distance of the PSF they appear as a single unresolved object. Even under optimal conditions the optical resolution of conventional fluorescence microscopy is limited to approximately half of the wavelength of the light used, which means that only objects separated by at least 200 to 350 nm can be resolved. This is the approximate size of intracellular organelles thus leaving fundamental biological processes beyond reach. Super-resolution techniques [208,209,210,211,212,213,214,215,216,217] overcome this limitation by activating the fluorophores in sequence so that adjacent molecules do not emit simultaneously. By effectively “breaking” the diffraction barrier it is possible to achieve image resolution of ~10 nm, which opens up whole new realms in plant biology [218,219,220].

With the increasing resolution of imaging technology and our enhanced understanding of processes within the cell, it is not impossible that a number of the basic assumptions underlying standard enzyme kinetics (and the models that use them) may need revisiting. The concept and validity of a concentration and its associated continuous change has already been shown to deviate from discrete models for small particle numbers and the importance of “stochasticity”, *i.e*., fluctuations due to low copy numbers, is beyond doubt. Likewise, the *in vitro* dilution ranges and relatives enzyme/ligand ratios for which the equations of enzyme kinetics were derived are far from physiological conditions. How diffusion varies locally in a crowded environment and how local changes in substrate and enzyme availability affect cellular processes is relatively poorly understood. In particular, for calcium signalling these local properties are likely to be of importance for both the encoding and decoding processes. It is not known how the physics of transport in temporally and spatially heterogeneous, crowded, viscous fluids might influence, for instance, channel gating, or other conformational changes. In the same way that concentrations become ill-defined for small compartments with low copy numbers, ionic currents, temperature and other macroscopic quantities that determine the behaviour of many models involving calcium may need reconsideration.

There is thus an enormous potential in these emerging experimental techniques to further the understanding of calcium signalling. The relevant components can only be imaged by super-resolution: e.g., the overall size of an IP_3_R channel is about 20 nm, with internal structures of the order of 2–3 nm [221], and the central pore of the nuclear pores complexes in plants is about 9–10 nm large [222]. Moreover, in some systems a calcium-release site of ~100–200 nm is actually a cluster comprising around 20–50 release channels that are distributed more closely than the resolution of conventional microscopy. This leads to a hierarchy of calcium signals of differing magnitudes, from the release by a single channel up to the flux by a cluster and a global calcium wave. The transition between different levels has been the object of several models [223,224,225,226,227,228,229,230] that highlight important dimensions of calcium signalling, such as stochasticity or others. The applicability of these and other models to the plant field can only be ascertained by the confrontation with experimental results at the relevant scales [231,232,233,234,235]. Particularly fruitful methods may arise from the integration of different techniques: combining patch-clamp methods with super-resolution microscopy, Bhargava *et al*. [236] were able to resolve both the activity and localization of calcium release channels within a microdomain. In the intersection between modelling and techniques such as super-resolution and FCS lies an exciting opportunity for advancing the understanding of calcium signalling in plants.

## Supplementary Files

Supplementary File 1
