# Supplementary material for: Towards the Physics of Calcium Signalling in Plants
Source: Plants (Basel). 2013 Sep 27;2(4):541–88. doi: 10.3390/plants2040541 (PMC4844391; doi:10.3390/plants2040541)
Supplement: Supplementary File 1 [file plants-02-00541-s001.zip › plants-38790-supplementary/plants-38790-Table S1-final.docx]

**Table S1.** Summary of models.

| **Context or section** | **Species** | **Stimulus** | **Ca^2+^ signature** | **Compartments** | **Components** | **Model** | **Ref.** |
| --- | --- | --- | --- | --- | --- | --- | --- |
| Calcium machinery | *Eremosphaera viridis* | Sr^+^ | Repetitive spikes | Cytosol,  ER | Sr^2+^ and Ca^2+^ gated Ca^2+^ channel, ATPase | ODE | [[1](#_ENREF_1)] |
| Calcium machinery  Symbiosis | *Medicago truncatula* | Nod factor | Oscillations | Nucleus,  nuclear envelope  store | Voltage gated Ca^2+^ channel, ligand gated K^+^ channel, Ca^2+^ pump | ODE | [[2](#_ENREF_2)]  [[3](#_ENREF_3)] |
| Calcium machinery  Temperature response | Tobacco Bright Yellow-2 (BY-2) | pH Temperature Mechanical stimulus | pH 7+, cold shock lowers [Ca^2+^]_nuc,_ while the resting levels increase with temperature  pH <7, repetitive mechanical stimulation elicits periodic stable variation of [Ca^2+^]_nuc_ | Nucleus,  nuclear envelope | Buffers, channels, pumps | ODE | [[4](#_ENREF_4)] |
| Calcium machinery  Symbiosis | *Medicago truncatula* | Nod factor | Oscillations | Nucleus,  cytosol | Pores, CICR channels, linear uptake by pumps | FDF | [[5](#_ENREF_5)] |
| Calcium machinery | *Arabidopsis thaliana* | Ca^2+^-CaM | Stable basal Ca^2+^ levels until a Ca^2+^ threshold where steep activation of the pump occurs | n/a | ATPase | Mass-action kinetics,ODEs | [[6](#_ENREF_6)] |
| Calcium machinery | *Beta vulgaris* L. | (1) Ca^2+^ (2) Mg^2+^ (3) Voltage | (1) Increases single and macroscopic current (2) Increases single-channel current (3) Biphasic increase | Vacuole | Slow vacuolar (SV) channels | Kinetic | [[7](#_ENREF_7)] |
| Response to temperature | Arabidopsis | Rapid cooling | Single [Ca^2+^]_cyt_ peak or biphasic response | Cytosol | Channels and pumps | State model | [[8](#_ENREF_8)] |

**Table S1.** *Cont*.

| **Context or section** | **Species** | **Stimulus** | **Ca^2+^ signature** | **Compartments** | **Components** | **Model** | **Ref.** |
| --- | --- | --- | --- | --- | --- | --- | --- |
| Response to temperature | Arabidopsis | Cold Osmotic Touch Anoxia | Oscillations with decreasing amplitude | Cytosol,  intracellular store | CAX, ATPase, two channels, buffers | ODE | [[9](#_ENREF_9)] |
| Response to temperature | *Secale cereale* L | Rapid cooling  Voltage | Single peak | Cytosol | Voltage-dependent “maxi” cation channel | 3B2S permeation, Kinetic | [[10](#_ENREF_10)] |
| Response to temperature | Arabidopsis | Low temperature/  pH | Single [Ca^2+^]_cyt_ peak | Vacuole,  cytosol | CICR channels, H^+^, K^+^, Cl^-^transporters | ODE | [[11](#_ENREF_11)] |
| Symbiosis | *Medicago truncatula* | Nod factor | Oscillations | Nucleus,  nuclear envelope  store | Ligand gated Ca^2+^ channel, Ca^2+^ pump | CWC | [[12](#_ENREF_12)] |
| Pollen tube growth | Lilium longiflorum | Growth, external calcium | Oscillations | Cytosol,  cell wall | fluid dynamics, viscoelastics, biomechanics, diffusion, turgor, stretch-activated channels | PDE | [[13](#_ENREF_13),14] |
| Pollen tube growth | Arabidopsis | Growth, external ions, Gradient | Oscillations | Cytosol tip  & shank | voltage gated calcium channels, ion transporters, pumps | ODE | [[15](#_ENREF_15)] |
| Pollen tube growth | Tobacco | Growth | Oscillations | Cytosol | actin, ROP1 | DDE | [[16](#_ENREF_16)] |
| Stomata | Arabidopsis | Various | N/A | Implicit from included processes | Implicit | Network | [[17](#_ENREF_17)] |

**Table S1.** *Cont*.

| **Context or section** | **Species** | **Stimulus** | **Ca^2+^ signature** | **Compartments** | **Components** | **Model** | **Ref.** |
| --- | --- | --- | --- | --- | --- | --- | --- |
| Stomata | *Commelina* | ABA | Oscillations | ER and tonoplast | Channels, pumps | ODE | [[18](#_ENREF_18)] |
| Stomata | *Vicia* | N/A | N/A | Apoplast,  Cytosol,  Vacuole | pH, channels, pumps | OnGuard | [[19](#_ENREF_19)] |
| Stomata | *Vicia* | Diurnal cycle | Varies diurnally | Apoplast,  Cytosol,  Vacuole | pH, channels, pumps | OnGuard | [[20](#_ENREF_20)] |
| Circadian rhythms | Arabidopis | Light | Oscillations | Cytosol | Phase delays | Systems Identification with time-invariant models | [[21](#_ENREF_21)] |
| Action Potential | Wheat | Rapid cooling | Voltage Spike, Ca^2+^ flux | Cytoplasm,  Apoplast | Voltage Ca^2+^, K^+^ channels, Ca^2+^ gated Cl^-^ channel, pumps, ion transporters | ODE | [[22](#_ENREF_22)] |
| Action Potential | Wheat | Rapid cooling | Propagating voltage spike, Ca^2+^ flux | Cytoplasm,  Apoplast |  | ODE | [[23](#_ENREF_23)] |
| Variation Potential | Wheat | Wounding | Propagating voltage spike, Ca^2+^ flux | Cytoplasm,  Apoplast | Ligand gated Ca^2+^ channel, as in previous | ODE | [[24](#_ENREF_24)] |
| Specificity | *Medicago truncatula* | Nod & Myc factors | Oscillations | Root hair cells | N/A | Bayesian spectral analysis | [[25](#_ENREF_25)]  [[26](#_ENREF_26)] |
| Specificity | *Medicago truncatula* | Nod & Myc factors | Oscillations | Root hair cells | N/A | Tests for deterministic chaos | [[27](#_ENREF_27)]  [[28](#_ENREF_28)] |
| Specificity | Arabidopsis | Temperature | Oscillations | Guard cells | N/A | Summation | [[29](#_ENREF_29)] |

**Table S1.** *Cont*.

| **Context or section** | **Species** | **Stimulus** | **Ca^2+^ signature** | **Compartments** | **Components** | **Model** | **Ref.** |
| --- | --- | --- | --- | --- | --- | --- | --- |
| Specificity | N/A | N/A | Single peak, global and local | Cells | N/A | CA | [[30](#_ENREF_30)] |
| Specificity - decoding | *V. Faba* | N/A | Train of square-shaped spikes | N/A | Proteins | ODE | [[31](#_ENREF_31)] |

References

1. Bauer, C.S.; Plieth, C.; Bethmann, B.; Popescu, O.; Hansen, U.P.; Simonis, W.; Schonknecht, G. Strontium-induced repetitive calcium spikes in a unicellular green alga. *Plant Physiol.* **1998**, *117*, 545–557.

2. Granqvist, E.; Wysham, D.; Hazledine, S.; Kozlowski, W.; Sun, J.; Charpentier, M.; Martins, T.V.; Haleux, P.; Tsaneva-Atanasova, K.; Downie, J.A.; Oldroyd, G.E.; Morris, R.J. Buffering capacity explains signal variation in symbiotic calcium oscillations. *Plant Physiol.* **2012**, *160*, 2300–2310.

3. Charpentier, M.; Vaz Martins, T.; Granqvist, E.; Oldroyd, G.E.; Morris, R.J. The role of DMI1 in establishing Ca(2+) oscillations in legume symbioses. *Plant Signal. Behav.***2013**, *8*, pii: e22894.

4. Briere, C.; Xiong, T.C.; Mazars, C.; Ranjeva, R. Autonomous regulation of free Ca2+ concentrations in isolated plant cell nuclei: A mathematical analysis. *Cell Calcium* **2006**, *39*, 293–303.

5. Capoen, W.; Sun, J.; Wysham, D.; Otegui, M.S.; Venkateshwaran, M.; Hirsch, S.; Miwa, H.; Downie, J.A.; Morris, R.J.; Ane, J.M.; Oldroyd, G.E. Nuclear membranes control symbiotic calcium signaling of legumes. *Proc. Natl. Acad. Sci. USA* **2011**, *108*, 14348–14353.

6. Tidow, H.; Poulsen, L.R.; Andreeva, A.; Knudsen, M.; Hein, K.L.; Wiuf, C.; Palmgren, M.G.; Nissen, P. A bimodular mechanism of calcium control in eukaryotes. *Nature* **2012**, *491*, 468–472.

7. Pottosin, I.I.; Martinez-Estevez, M.; Dobrovinskaya, O.R.; Muniz, J.; Schönknecht, G. Mechanism of luminal Ca2+ and Mg2+ action on the vacuolar slowly activating channels. *Planta* **2004**, *219*, 1057–1070.

8. Plieth, C. Temperature sensing by plants: calcium-permeable channels as primary sensors—A model. *J. Membr. Biol.* **1999**, *172*, 121–127.

9. Bose, J.; Pottosin, I.I.; Shabala, S.S.; Palmgren, M.G.; Shabala, S. Calcium efflux systems in stress signaling and adaptation in plants. *Front Plant Sci.* **2011**, *2*, 85.

10. White, P.J.; Ridout, M.S. An energy-barrier model for the permeation of monovalent and divalent cations through the maxi cation channel in the plasma membrane of rye roots. *J. Membr. Biol.* **1999**, *168*, 63–75.

11. Liu, J.; Knight, H.; Hurst, C.H.; Knight, M.R. Modelling and experimental analysis of the role of interacting cytosolic and vacuolar pools in shaping low temperature calcium signatures in plant cells. *Mol. Biosyst.* **2012**, *8*, 2205–2220.

12. Sciacca, E.; Spinella, S.; Genre, A.; Calcagno, C. Analysis of Calcium Spiking in Plant Root Epidermis through CWC Modeling. *Electron. Notes Theor. Comput. Sci.* **2011**, *277*, 65–76.

13. Kroeger, J.H.; Geitmann, A.; Grant, M. Model for calcium dependent oscillatory growth in pollen tubes. *J. Theor. Biol.* **2008**, *253*, 363–374.

14. Kroeger, J.H.; Zerzour, R.; Geitmann, A. Regulator or driving force? The role of turgor pressure in oscillatory plant cell growth. *PLoS One* **2011**, *6*, e18549.

15. Liu, J.; Piette, B.M.; Deeks, M.J.; Franklin-Tong, V.E.; Hussey, P.J. A compartmental model analysis of integrative and self-regulatory ion dynamics in pollen tube growth. *PLoS One* **2010**, *5*, e13157.

16. Yan, A.; Xu, G.; Yang, Z.B. Calcium participates in feedback regulation of the oscillating ROP1 Rho GTPase in pollen tubes. *Proc. Natl. Acad. Sci. USA* **2009**, *106*, 22002–22007.

17. Li, S.; Assmann, S.M.; Albert, R. Predicting essential components of signal transduction networks: A dynamic model of guard cell abscisic acid signaling. *PLoS Biol.* **2006**, *4*, e312.

18. Veresov, V.G.; Kabak, A.G.; Volotovsky, I.D. Modeling the calcium signaling in stomatal guard cells under the action of abscisic acid. *Russ. J. Plant Physiol.* **2003**, *50*, 573–579.

19. Hills, A.; Chen, Z.H.; Amtmann, A.; Blatt, M.R.; Lew, V.L. OnGuard, a computational platform for quantitative kinetic modeling of guard cell physiology. *Plant Physiol.* **2012**, *159*, 1026–1042.

20. Chen, Z.H.; Hills, A.; Batz, U.; Amtmann, A.; Lew, V.L.; Blatt, M.R. Systems dynamic modeling of the stomatal guard cell predicts emergent behaviors in transport, signaling, and volume control. *Plant Physiol.* **2012**, *159*, 1235–1251.

21. Dalchau, N.; Hubbard, K.E.; Robertson, F.C.; Hotta, C.T.; Briggs, H.M.; Stan, G.B.; Goncalves, J.M.; Webb, A.A.R. Correct biological timing in Arabidopsis requires multiple light-signaling pathways. *Proc. Natl. Acad. Sci. USA* **2010**, *107*, 13171–13176.

22. Sukhov, V.; Vodeneev, V. A mathematical model of action potential in cells of vascular plants. *J. Membr. Biol.* **2009**, *232*, 59–67.

23. Sukhov, V.; Nerush, V.; Orlova, L.; Vodeneev, V. Simulation of action potential propagation in plants. *J. Theor. Biol.* **2011**, *291*, 47–55.

24. Sukhov, V.; Akinchits, E.; Katicheva, L.; Vodeneev, V. Simulation of variation potential in higher plant cells. *J. Membr. Biol.* **2013**, *246*, 287–296.

25. Granqvist, E.; Oldroyd, G.E.; Morris, R.J. Automated Bayesian model development for frequency detection in biological time series. *BMC Syst. Biol.* **2011**, *5*, 97.

26. Granqvist, E.; Hartley, M.; Morris, R.J. BaSAR-A tool in R for frequency detection. *Biosystems* **2012**, *110*, 60–63.

27. Kosuta, S.; Hazledine, S.; Sun, J.; Miwa, H.; Morris, R.J.; Downie, J.A.; Oldroyd, G.E. Differential and chaotic calcium signatures in the symbiosis signaling pathway of legumes. *Proc. Natl. Acad. Sci. USA* **2008**, *105*, 9823–9828.

28. Hazledine, S.; Sun, J.; Wysham, D.; Downie, J.A.; Oldroyd, G.E.; Morris, R.J. Nonlinear time series analysis of nodulation factor induced calcium oscillations: evidence for deterministic chaos? *PLoS One* **2009**, *4*, e6637.

29. Dodd, A.N.; Jakobsen, M.K.; Baker, A.J.; Telzerow, A.; Hou, S.W.; Laplaze, L.; Barrot, L.; Poethig, R.S.; Haseloff, J.; Webb, A.A. Time of day modulates low-temperature Ca signals in Arabidopsis. *Plant J.* **2006**, *48*, 962–973.

30. Plieth, C. Signal percolation through plants and the shape of the calcium signature. *Plant Signal. Behav.* **2010**, *5*, 379–385.

31. Marhl, M.; Perc, M.; Schuster, S. A minimal model for decoding of time-limited Ca2+ oscillations. *Biophys. Chem.* **2006**, *120*, 161–167.
