# Supplementary material for: Towards the Physics of Calcium Signalling in Plants
Source: Plants (Basel). 2013 Sep 27;2(4):541–88. doi: 10.3390/plants2040541 (PMC4844391; doi:10.3390/plants2040541)
Supplement: Supplementary File 1 [file plants-02-00541-s001.zip › plants-38790-supplementary/plants-38790-Table S2-final.docx]

**Table S2.** Alphabetical list of resources for the visualisation and modelling of plant cell processes.

| **Software/tool** | **Website** | **Description** |
| --- | --- | --- |
| BaSAR [1] | cran.r-project.org/web/packages/BaSAR | Bayesian Spectrum Analysis in R—analysis of time series data. |
| Bio-PEPA [2] | http://www.biopepa.org/ | Modelling and the analysis of biochemical networks*.* |
| COPASI | www.copasi.org | Simulate and analyse biochemical networks. |
| CWC simulators | scwc-sim.sourceforge.net | Simulation software implementing the Calculus of Wrapped Compartments (CWC). |
| E-Cell [3] | www.e-cell.org | Software to simulate the whole cell down to the molecular level. |
| HoTSig [4] | www.psrg.org.uk | Software libraries for the modelling of compartmentalised kinetically-driven systems. |
| iPlant | www.iplantcollaborative.org | Collaborative effort funded by the NSF to develop the cyberinfrastructure required by all plant sciences. |
| LENNS | www.eeb.cornell.edu/Ellner/software.html | Lyapunov Exponents for Noisy Nonlinear Systems—analysis tool written in R. |
| Matlab | www.mathworks.co.uk/products/matlab | Computational and visualisation workbench. |
| MaxChelator | www.stanford.edu/~cpatton | Estimates free metal ion concentration in the presence of chelators. |
| Octave | www.gnu.org/software/octave | Computational and visualisation workbench (similar to Matlab). |
| OnGuard [4] | www.psrg.org.uk | Model of a stomate—based on HoTSig. |
| Plant Image Analysis | www.plant-image-analysis.org | Repository of ~90 published image analysis tools for plants. |
| SBML | sbml.org | Systems Biology Markup Language—a text-based format for representing models. |
| SBW | sbw.sourceforge.net | Systems Biology Workbench—Integrates models via CellML (www.cellml.org) and SBML. |
| TISEAN [5] | www.mpipks-dresden.mpg.de/~tisean/Tisean_3.0.1/index.html | Nonlinear time series analysis tool. |
| VCell [6] | www.nrcam.uchc.edu | Distributed computational environment for modelling cell biology. Parameter estimation uses COPASI. |
| XPP-Aut | www.math.pitt.edu/~bard/bardware/xpp/xpp.html | Differential, delay and stochastic equation solver with bifurcation analysis |

References

1. Granqvist, E.; Hartley, M.; Morris, R.J. BaSAR—A tool in R for frequency detection. *BioSystems* **2012**, *110*, 60–63.
2. Ciocchetta, F.; Hillston, J. Bio-PEPA: A Framework for the Modelling and Analysis of Biochemical Networks. *Theor. Comput. Sci.* **2009**, *410*, 3065–3084.
3. Tomita, M.; Hashimoto, K.; Takahashi, K.; Shimizu, T.; Matsuzaki, Y.; Miyoshi, F.; Saito, K.; Tanida, S.; Yugi, K.; Venter, J.C.; Hutchison, C.A.
   E-CELL: Software environment for whole cell simulation. *Genome Inform. Ser. Workshop Genome Inform.* **1997**, *8*, 147–155.
4. Hills, A.; Chen, Z.H.; Amtmann, A.; Blatt, M.R.; Lew, V.L. OnGuard, a computational platform for quantitative kinetic modeling of guard cell physiology. *Plant Physiol.* **2012**, *159*, 1026-1042.
5. Hegger, R.; Kantz, H.; Schreiber, T. Practical implementation of nonlinear time series methods: The TISEAN package. *Chaos* **1999**, *9*, 413.
6. Schaff, J.; Fink, C.C.; Slepchenko, B.; Carson, J.H.; Loew, L.M. A general computational framework for modeling cellular structure and function. *Biophys. J.* **1997**, *73*, 1135–1146.
